# A Flat BAR Protein Promotes Actin Polymerization at the Base of Clathrin-Coated Pits

**DOI:** 10.1016/j.cell.2018.05.020

**Published:** 2018-07-12

**Authors:** Leonardo Almeida-Souza, Rene A.W. Frank, Javier García-Nafría, Adeline Colussi, Nushan Gunawardana, Christopher M. Johnson, Minmin Yu, Gillian Howard, Byron Andrews, Yvonne Vallis, Harvey T. McMahon

**Affiliations:** 1 MRC Laboratory of Molecular Biology, Francis Crick Avenue, Cambridge CB2 0QH, UK

**Keywords:** clathrin-mediated endocytosis, actin cytoskeleton, membrane deformation, FCHSD2, BAR domain, Nervous Wreck, intersectin, N-WASP activation, ARP2/3, cytoskeletal forces

## Abstract

Multiple proteins act co-operatively in mammalian clathrin-mediated endocytosis (CME) to generate endocytic vesicles from the plasma membrane. The principles controlling the activation and organization of the actin cytoskeleton during mammalian CME are, however, not fully understood. Here, we show that the protein FCHSD2 is a major activator of actin polymerization during CME. FCHSD2 deletion leads to decreased ligand uptake caused by slowed pit maturation. FCHSD2 is recruited to endocytic pits by the scaffold protein intersectin via an unusual SH3-SH3 interaction. Here, its flat F-BAR domain binds to the planar region of the plasma membrane surrounding the developing pit forming an annulus. When bound to the membrane, FCHSD2 activates actin polymerization by a mechanism that combines oligomerization and recruitment of N-WASP to PI(4,5)P_2_, thus promoting pit maturation. Our data therefore describe a molecular mechanism for linking spatiotemporally the plasma membrane to a force-generating actin platform guiding endocytic vesicle maturation.

## Highlights

• FCHSD2 is a bona fide CME protein recruited to CCPs by intersectin
• Intersectin recruits FCHSD2 via an SH3-SH3 interaction
• FCHSD2 is a major activator of actin during CME
• FCHSD2 binds to the surrounding membrane around CCPs via its flat F-BAR domain

## Graphical Abstract

**Figure:**
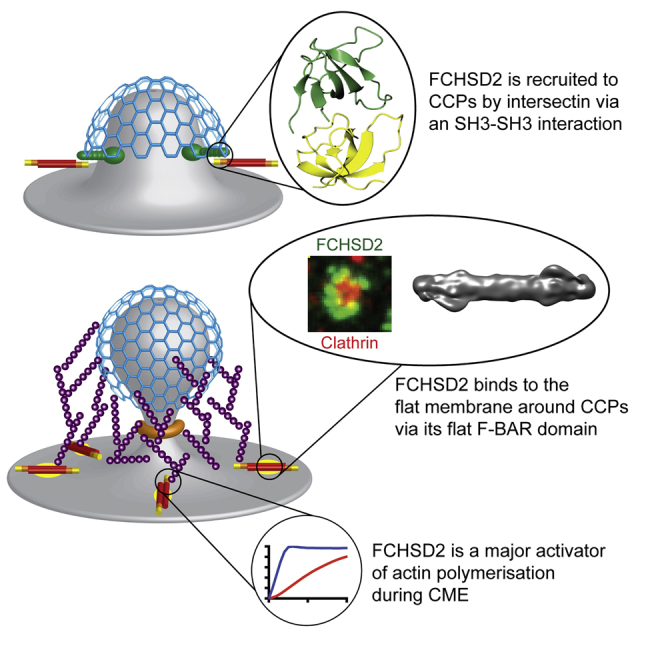

## Teaser

A flat BAR protein binds to the membrane surrounding clathrin-coated pits and promotes actin polymerization to aid endocytic vesicle maturation.

## Introduction

Clathrin-mediated endocytosis (CME) is a process by which cells internalize receptors, nutrients, lipids, and pathogens. CME is characterized by the formation of dome-shaped membrane invaginations covered by a polygonal clathrin cage termed clathrin-coated pits (CCPs). A complex protein machinery works alongside clathrin for initiation, growth, and scission of the CCP. Despite being an ever present feature of CCPs (Ferguson et al., 2009, Grassart et al., 2014), how the actin cytoskeleton affects the process remains a matter of debate as the requirement for the mechanical forces provided by this cytoskeletal component seems to vary depending on cell type, membrane tension, and cargo (Boulant et al., 2011, Fujimoto et al., 2000). Over the years, multiple actin regulators have been described to participate in CME, including many BAR domain proteins (Daumke et al., 2014, Merrifield and Kaksonen, 2014). The actin cytoskeleton at CCPs is organized as a densely branched network that evolves from lateral patches around shallow pits to a “comet tail” structure at the final stages of endocytosis (Collins et al., 2011, Salisbury et al., 1980). Significantly, the actin cables on CCPs are polymerized from the plasma membrane toward the clathrin cage, supporting the idea that actin polymerization helps to propel the CCP inward (Collins et al., 2011, Picco et al., 2015). How this special actin organization arises and what the relative contribution is of each one of the different actin regulators described for CME remains largely unknown.

FCH and double SH3 domains protein 1 and 2 (FCHSD1 and FCHSD2) are the mammalian homologs of the *Drosophila* Nervous Wreck protein (Nwk). They are part of the BAR superfamily of dimeric membrane binding domains (https://www.bar-superfamily.org). Nwk mutant flies are paralyzed under non-permissive temperatures and show abnormal neuronal morphology (Coyle et al., 2004). The Nwk protein interacts with components of the CME and actin cytoskeleton machinery (O’Connor-Giles et al., 2008, Rodal et al., 2008), but a detailed understanding of its function, or of its mammalian homologs FCHSD1/2, remains elusive. Here, we show that FCHSD2 is a major activator of actin polymerization during CME. FCHSD2 is recruited to CCPs by intersectin via an SH3-SH3 interaction and localizes to the base of CCPs where it activates actin polymerization via N-WASP.

## Results

**Figure 1:**
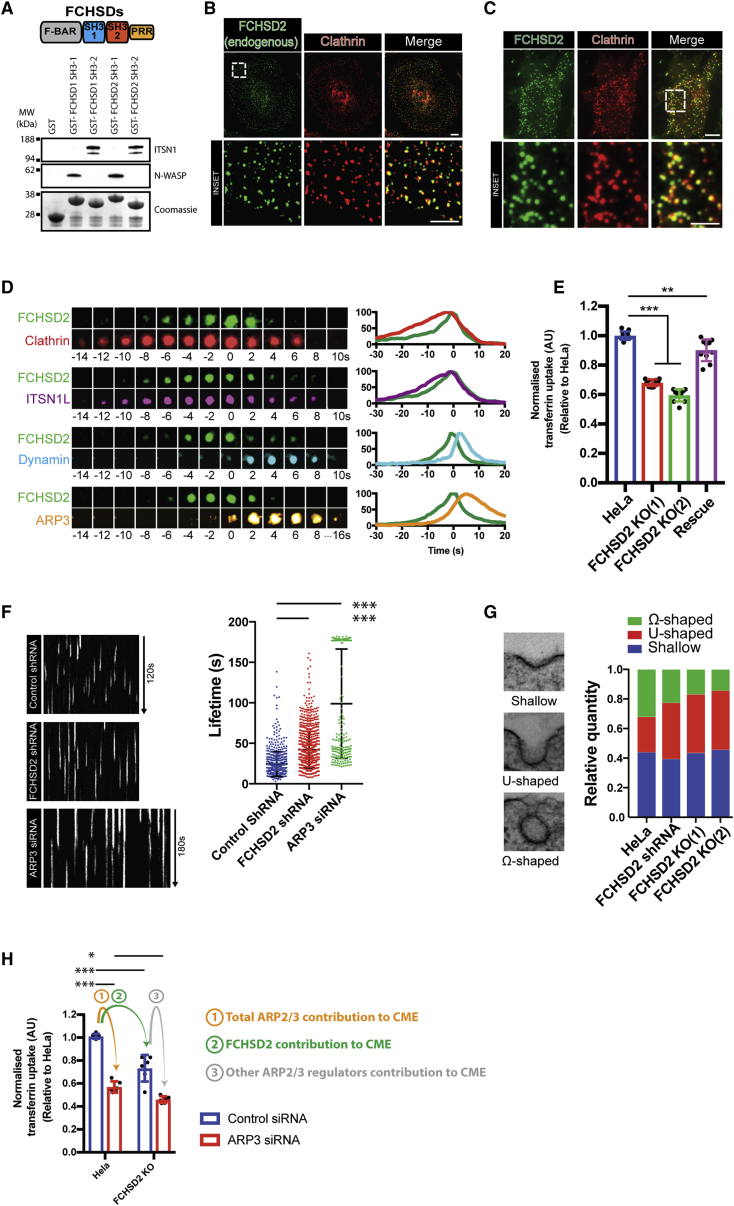
FCHSD2 Is a Bona Fide CME Protein Responsible for a Major Fraction of the ARP2/3 Contribution to CME (A) Top: Scheme showing the domain organization of FCHSD proteins. Bottom: Immunoblots for N-WASP and Intersectin1 (ITSN1) from pull down experiments from brain extracts using GST-tagged FCHSD1 and FCHSD2 SH3 domains. Lower portion shows Coomassie staining of baits. (B) Immunofluorescence showing colocalization between endogenous FCHSD2 and clathrin heavy chain. (C) TIRF image showing colocalization of FCHSD2 and clathrin. HeLa cells stably expressing FCHSD2-Venus and transfected with mCherry-clathrin light chain. (D) Left: Examples of the dynamics of FCHSD2 with different CME proteins. HeLa cells stably expressing FCHSD2-Venus were transfected with mCherry-clathrinLC, FusionRed-ITSN1L, FusionRed-Dynamin1, or mCherry-ARP3 and imaged live by TIRF microscopy. Time zero was set as the peak of FCHSD2 recruitment. Events are pseudocolored to match graphs on the right. Right: Summary graphs for the timing of recruitment of FCHSD2 versus CME proteins (n = 90, 48, 120, and 144 events for FCHSD2/clathrin, FCHSD2/ITSN1L, FCHSD2/Dynamin, and FCHSD2/ARP3, respectively). Full data including error bars are shown in Figure S1A. (E) Transferrin uptake assay by flow cytometry. Uptake measurements were normalized as described in STAR Methods. Each value represents median fluorescence from at least 5,000 cells (n = 10, mean ± SD). (F) Left: Kymographs of BSC1 AP2σ2-GFP cells silenced for FCHSD2 or ARP3 and control cells. Kymographs generated from 120 s videos at 1 Hz (or 180 s at 1 Hz in the case of ARP3 small interfering RNA [siRNA] cells). Right: Quantification of AP2σ2 lifetime for each condition. Only events longer than 20 s were considered (n = 329, 870, and 227 events for control, FCHSD2 KD, and ARP3 KD, respectively, mean ± SD). (G) CCP morphological quantification for control HeLa and FCHSD2 KD and KO cells (n = 100, 71, 101, and 70 CCPs for control, shRNA, KO(1), and KO(2) cells, respectively). (H) Transferrin uptake assay by flow cytometry comparing wild-type and FCHSD2 KO (2) cells silenced for ARP3. Uptake measurements were normalized as described in the STAR Methods. Each value represents median fluorescence from at least 5,000 cells (n = 6, mean ± SD). ^∗∗∗^p > 0.001, ^∗∗^p > 0.01, ^∗^p > 0.01, one-way ANOVA with Tukey’s post hoc analysis. Scale bars, 10 μm in overviews, 5 μm in insets. See also Figures S1 and S2 and Videos S1 and S2 and Videos S1 and S2.

Vertebrate genomes encode two FCHSD proteins (FCHSD1 and FCHSD2) that contain 4 distinct domains as shown in Figure 1A: (1) an N-terminal F-BAR domain containing an atypical additional coiled coil (CC) at its C terminus, (2) a first SH3 (src homology 3) domain (SH3-1), (3) a second SH3 domain (SH3-2), and (4) a C-terminal proline rich region (PRR). GST pull downs from brain extracts using individual SH3 domains as bait confirmed that FCHSD1/2, like its fly homolog Nwk (O’Connor-Giles et al., 2008, Rodal et al., 2008), interact with N-WASP and intersectin via its SH3-1 and SH3-2, respectively (Figure 1A). FCHSD1 is generally expressed at lower levels than FCHSD2 (Uhlén et al., 2015). Moreover, FCHSD1 is not detectable in the cells lines we worked with (Hein et al., 2015). We therefore focused on the main isoform FCHSD2.

### FCHSD2 Is a Bona Fide CME Protein

**Figure S1:**
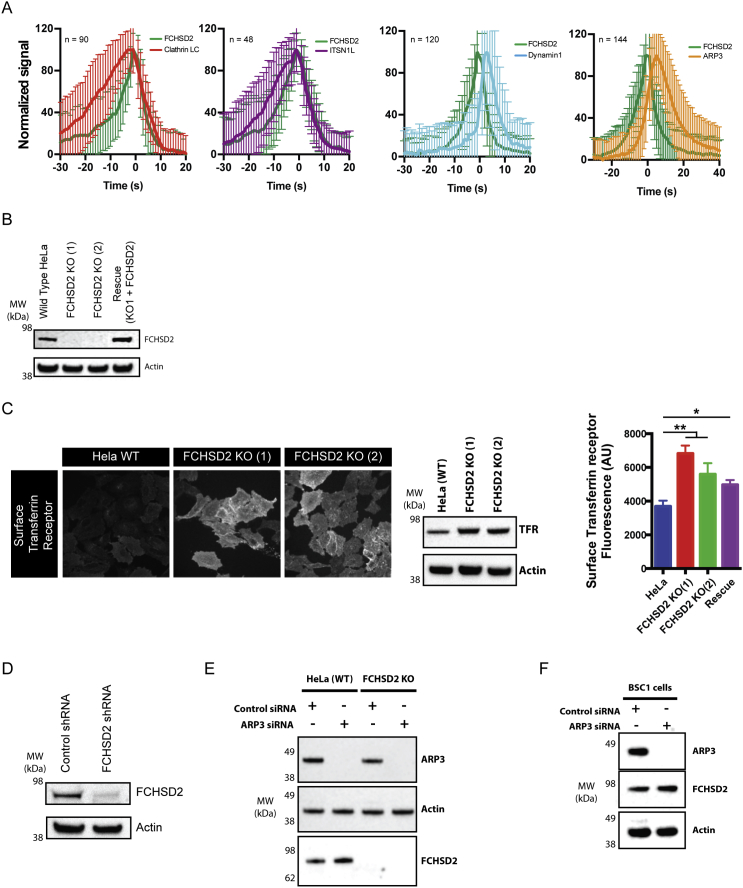
FCHSD2 Is a Bona Fide CME Protein, Related to Figure 1 (A) Graphs for the recruitment dynamics of FCHSD2 versus ClathrinLC, Dynamin1, ITSN1L and ARP3. Number of events measured are shown at the top left of each graph. Data are presented as mean ± SD. (B) Immunoblots for FCHSD2 showing both FCHSD2 CRISPR/Cas9 knockout clones used and the rescue cell line (FCHSD2 KO-1 stably expressing untagged FCHSD2). (C) Increased surface and total transferrin receptor (TFR) in FCHSD2 knockout cells shown by immunofluorescence (left), immunoblots (center) and flow cytometry (right). Each value represents median fluorescence from at least 5000 cells (n = 3, mean ± SD). ^∗∗^p > 0.01, ^∗^p > 0.05. One-way ANOVA with Tukey’s post hoc analysis. (D) Immunoblots showing FCHSD2 knockdown in BSC1 AP2σ2-GFP cells. (E) Immunoblots showing ARP3 knockdown in wild-type and FCHSD2 KO cells. (F) Immunoblots showing ARP3 knockdown in BSC1 AP2σ2-GFP cells.

The interaction of FCHSD2 and Nwk with CME components is the primary indication of their participation in this process (O’Connor-Giles et al., 2008, Rodal et al., 2008). To investigate this matter further, we started by looking at the subcellular localization and the dynamics of FCHSD2 during CME. Endogenous FCHSD2 showed clear colocalization to clathrin punctae on the plasma membrane (Figure 1B). Likewise, exogenously expressed FCHSD2-Venus formed dynamic punctae colocalizing with the majority of mCherry-clathrin punctae (mCherry-clathrin light chain A) (80% ± 7% measured over the period of 10 s, n = 11 cells and Figure 1C). Live-cell imaging using a series of CME components revealed that FCHSD2 recruitment to CCPs occurred at a mid-to-late stage of pit maturation. FCHSD2 signal appeared on CCPs after clathrin and intersectin and peaked before the recruitment of dynamin and ARP3 (Figures 1D and S1A; Video S1). Further confirming the participation of FCHSD2 in CME, FCHSD2 knockout (KO) cells displayed reduced transferrin uptake when compared with control cells (Figures 1E and S1B). Interestingly, FCHSD2 KO cells compensate for slowed endocytosis by increasing the amount of total and surface transferrin receptor (Figure S1C). This compensation phenomenon has also been reported for cells depleted for PI3KC2α, epsin, or dynamin (Ferguson et al., 2009, Messa et al., 2014, Posor et al., 2013). Re-expression of FCHSD2 could partially rescue the transferrin uptake defect (Figures 1E and S1B). We attribute this partial rescue to variations in expression level within the population of rescued cells.

Video S1. FCHSD2 Is a CME Protein Recruited to CCPs before Scission, Related to Figure 1 TIRF movies of HeLa cells stably expressing FCHSD2-Venus (in green) and various CME proteins mCherry-ClathrinLC, FusionRed-ITSN1L, FusionRed-Dynamin1 or mCherry-ARP3 (all in red).

**Figure S2:**
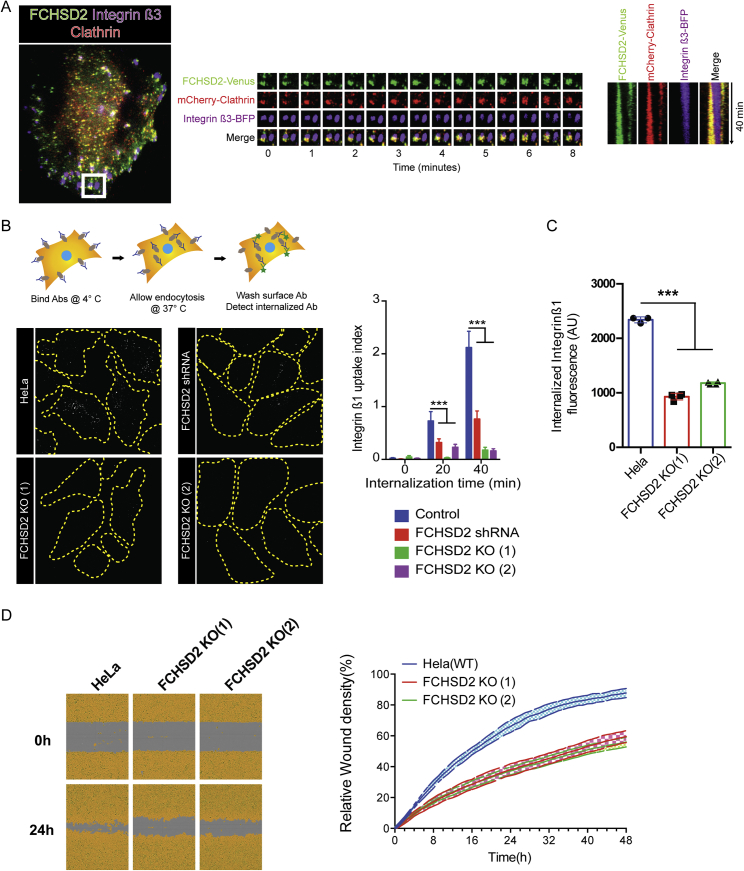
FCHSD2 Depletion Reduces Integrin Uptake and Affects Cell Migration, Related to Figure 1 (A) FCHSD2 localizes to disassembling focal adhesions. Left: Overview of cell expressing FCHSD2-Venus, mCherry-ClathrinLC and Integrin β3-BFP. Center: Image series of region marked in (left). The accumulation of FCHSD2 and clathrin occurs only at disassembling focal adhesions. Right: Kymograph showing FCHSD2 and clathrin advancing in a disassembling focal adhesion. Image series generated at 0.5 frame/minute. (B) FCHSD2 depletion reduces integrin β1 uptake. Top left: pictorial explanation of the experimental design used for the antibody (Ab)-feeding assay. Bottom left: representative images of the antibody feeding assay results. Cell boundaries are shown in yellow and internalised antibody signal is shown in white. Right: quantification of the antibody feeding assay using an antibody recognizing an active form of Integrin β1 (12G10). n = 27, 56, 53 (control); 27, 59, 80 (shRNA); 16, 61, 60 (FCHSD2 KO-1); 43, 51, 71 (FCHSD2 KO-2), mean ± SD. ^∗∗∗^p > 0.001, ^∗∗^p > 0.01. One-way ANOVA with Tukey’s post hoc analysis. (C) Integrin β1 uptake experiment performed by flow cytometry. For this experiment we used labeled Integrin β1 (12G120) antibody (Alexa 488). Each value represents median fluorescence from at least 5000 cells (n = 4, mean ± SD). ^∗∗∗^p > 0.001, ^∗∗^p > 0.01, ^∗^p > 0.05. One-way ANOVA with Tukey’s post hoc analysis. (D) Wound healing migration assay. Left: Representative images of control and knockout cells at time zero and at 24hs after wounding. Cell area is masked in yellow. Right: quantification of wound area closure over time (mean ± SD).

In several of our live cell experiments, we noticed the accumulation of FCHSD2 punctae on the peripheral regions of moving cells resembling adhesion sites. This led us to look at the relationship of FCHSD2 and integrins, a CME cargo associated with focal adhesions (Ezratty et al., 2009). In cells simultaneously expressing integrin β3, clathrinLC, and FCHSD2, we observed the presence of dynamic FCHSD2 and clathrin punctae on the majority of disassembling focal adhesions (Figure S2A; Video S2). Accordingly, FCHSD2 KO and knockdown (KD) cells showed significant impairment of active integrinß1 internalization using a microscopy-based antibody-feeding assay (Figure S2B) and by flow cytometry (Figure S2C). In agreement with the integrin uptake defect, FCHSD2 knockout led to a significant reduction of cell migration as measured with a wound-healing assay (Figure S2D).

Video S2. FCHSD2 Colocalize with Clathrin in Disassembling Focal Adhesions, Related to Figure 1 Zoomed view of a HeLa cell transfected with FCHSD2-GFP, Integrin β3- BFP and mCherry-ClathrinLC.

To further investigate the role of FCHSD2 on CME, we tested the effect of FCHSD2 depletion on CME dynamics. For that, we used BSC1 cells stably expressing GFP-tagged σ2-adaptin (AP2σ2-GFP) (Ehrlich et al., 2004). As shown in Figure 1F, cells depleted for FCHSD2 (Figure S1D) displayed longer CCP lifetimes than control cells (44.9 s ± 21.7 s for FCHSD2 small hairpin RNA [shRNA] cells versus 32.7 s ± 16.3 s for control cells, mean ± SD). Morphological analysis of pits by electron microscopy (EM) revealed that FCHSD2 KD and KO resulted in an increased frequency of intermediate stage pits (U-shaped) at the expense of terminal pits (Ω-shaped) (Figure 1G). In agreement with the mid-to-late recruitment of FCHSD2 to CCPs (Figure 1D), we found no change on the frequency of early stage pits (shallow pits) in FCHSD2-depleted cells. Taken together, our results show that FCHSD2 is a bona fide CME component whose depletion leads to a kinetic defect in CME.

### FCHSD2 Accounts for a Large Part of the Actin Contribution to CME

The interaction of FCHSD2 with N-WASP (Figure 1A) suggests a participation of this protein controlling actin polymerization during CME. To understand what the relative importance of FCHSD2 to the actin component of CME is, we decided to evaluate the CME phenotype in cells on which knockdown had been performed for the essential subunit of the ARP2/3 complex, ARP3 (Gournier et al., 2001). This strategy allowed us to look specifically at the effect of branched actin, the main type of actin structure formed during CME (Collins et al., 2011), without affecting linear actin structures and circumventing typical actin poisons that are very toxic and were shown to only partially affect the actin around CCPs (Collins et al., 2011). Cells with ARP3 KD (Figure S1E) showed a substantial reduction (∼43%) in transferrin uptake (Figure 1H) and displayed a large increase in CCP lifetimes with around a third of events lasting at least 180 s (the entire duration of the movies) (Figure 1F). Similar to the effect of FCHSD2 KO, ARP3 KD also led to an increase in surface transferrin receptor (increase of 37% ± 15% compared to wild-type cells, n = 5 independent experiments with >5,000 cells).

Next, we compared the transferrin uptake of cells with single and combined ARP3 and FCHSD2 depletion. In accordance with FCHSD2 and ARP2/3 acting on CME via the same pathway with ARP2/3 as a downstream factor, cells with combined ARP3 KD and FCHSD2 KO showed a similar reduction in transferrin uptake as cells with ARP3 KD alone (Figure 1H). An incomplete KD may contribute to the small difference in transferrin uptake between ARP3 KD in wild-type and in FCHSD2 KO cells (p value = 0.04). Importantly, with ARP2/3 as a downstream factor of FCHSD2, we could use the ARP2/3 contribution to CME as a baseline to infer that FCHSD2 is a major contributor to the ARP2/3 participation in CME. (Figure 1H).

### FCHSD2 Is Recruited to CCPs by Intersectin via an SH3-SH3 Interaction

**Figure 2:**
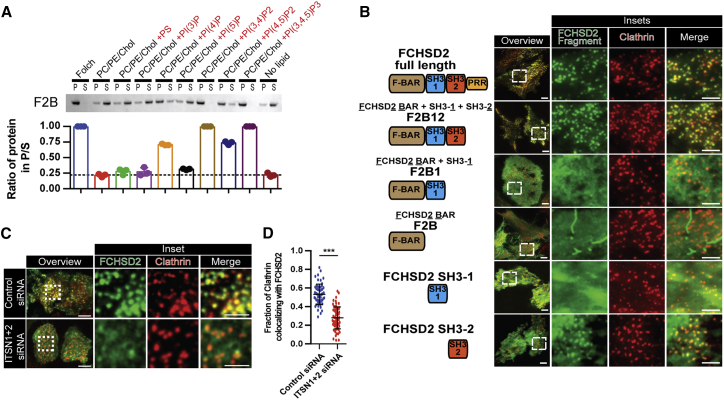
Intersectin Recruits FCHSD2 to CCPs (A) Lipid preference for FCHSD2 using liposome-pelleting assays. Liposome base mixture of PC, PE, and cholesterol was supplemented with PS or phosphoinositols (PIPs). After incubation with FCHSD2 BAR (F2B), the liposomes were pelleted by ultracentrifugation. P, pellet fraction; S, supernatant fraction. Dashed horizontal line represents the level of protein alone that pellets under the experimental conditions (n = 3 experiments, mean ± SD). (B) TIRF images of HeLa cells transfected with mCherry-clathrinLC and various GFP-tagged FCHSD2 truncation constructs. The domain arrangement for each construct with their respective nomenclature is shown on the left. (C) TIRF images of HeLa cells stably expressing FCHSD2-Venus and mCherry-clathrinLC transfected with a non-targeting control siRNA or siRNA for intersectins (ITSN1+ITSN2). (D) Quantification of the fraction of clathrin punctae colocalizing with FCHSD2 punctae on control and ITNS1+2 KD cells (n = 60, 75 cells for control siRNA and ITSN1+2 siRNA respectively; mean ± SD). ^∗∗∗^p > 0.001, t test. Scale bars, 10 μm in overviews, 5 μm in insets. See also Figure S3.

**Figure S3:**
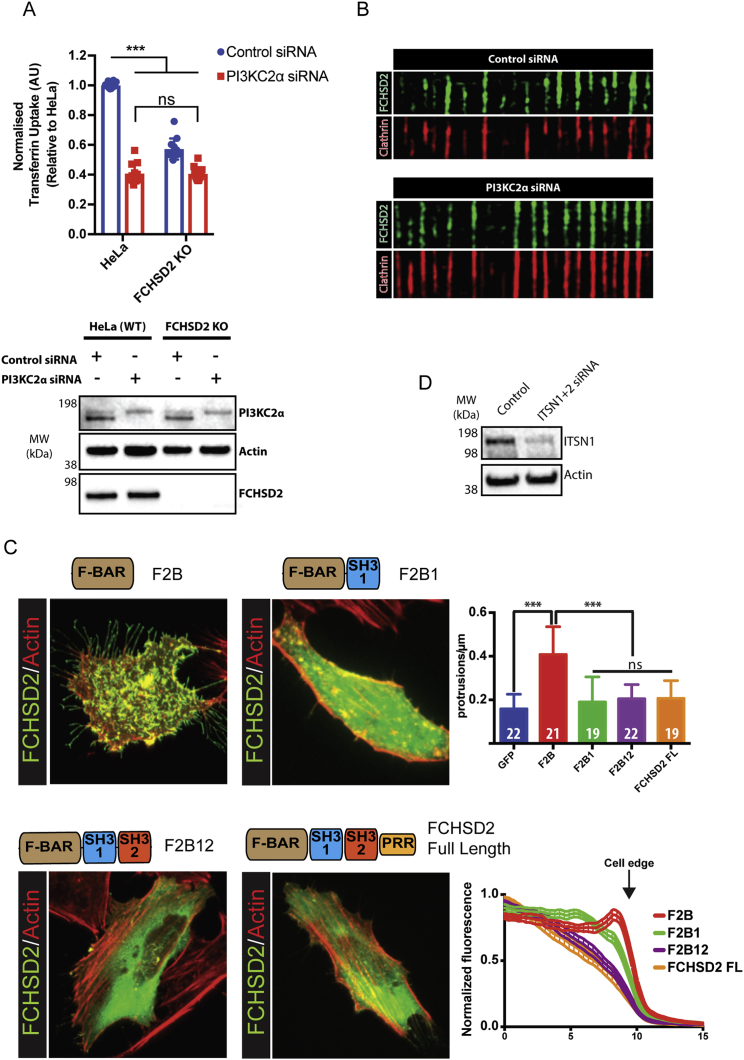
FCHSD2 Is Not Directly Recruited to CCPs by PI3KC2α or Its Kinase Activity, Related to Figure 2 (A) Top: Transferrin uptake assay by flow cytometry comparing wild-type and FCHSD2 KO cells silenced for PI3KC2α. Uptake of Alexa488 labeled transferrin normalized by the amount of surface transferrin receptor for each condition and against uptake for the wild-type cells in each experiment. Each value represents median fluorescence from at least 5000 cells (n = 12, mean ± SD). ^∗∗∗^p > 0.001, ns = non-significant. One-way ANOVA with Tukey’s post hoc analysis. Bottom: Immunoblots showing PI3KC2α knockdown in wild-type and FCHSD2 KO cells. (B) Kymographs of HeLa FCHSD2-Venus stables silenced for PI3KC2α and control cells. Cells were transfected with mCherry-ClathrinLC 24 hs before imaging. Kymographs generated from 120 s movies at 1Hz. Note the elongated CCP lifetimes in PI3KC2α knockdown cells as described in Posor et al. (2013). (C) Autoinhibition of FCHSD2. Representative images of a center slice from cells expressing different FCHSD2 truncation constructs and co-stained with phalloidin (Actin). The bar graph (upper right) shows the quantification of cellular protrusions/μm for each construct. The non-inhibited BAR domain produces many protrusions. Numbers inside bars represent number of cells measured. The line graph (bottom right) shows the fluorescence profile of sum intensity projections for cells expressing each construct. Due to the natural thinning of cells from their centers to the edge, a gradually decaying line indicates that the fluorescent protein is primarily cytosolic while a flat line with an abrupt fall on the cell edge indicates that the fluorescent protein is primarily bound to the membrane. While the presence of SH3-1 significantly reduces the generation of cellular protrusions generated by the FCHSD2 F-BAR, a significant fraction of the protein remains bound to the membrane (green line). Only the combined presence of SH3-1 and SH3-2 is capable to avoid promiscuous binding of the BAR domain to the membrane. Data is shown as mean ± SD in bar graph and as ± SEM in fluorescence profiles. ^∗∗∗^p > 0.001, ns = non-significant. One-way ANOVA with Tukey’s post hoc analysis. (D) Immunoblots for intersectin knockdown in FCHSD2-Venus HeLa cells.

CCP maturation is characterized by a cascade of phosphoinositide conversions that work as timekeepers of the process by regulating the recruitment of individual CME components (He et al., 2017, Posor et al., 2013). To identify if FCHSD2 has a preference for any specific phosphoinositide, and could explain its recruitment to CCPs, we used a liposome-pelleting assay with liposomes of specific compositions. As shown in Figure 2A, FCHSD2 binds preferentially to PI(3,4)P_2_ or PI(3,4,5)P_3_ containing liposomes. PI(3,4)P_2_ was recently shown to be a late CME lipid generated by PI3KC2α (Posor et al., 2013). In agreement with a potential role of PI(3,4)P_2_ on FCHSD2 function, KD of PI3KC2α showed similar decrease in transferrin uptake as the combined KD of PI3KC2α in FCHSD2 KO cells (Figure S3A). However, KD of PI3KC2α had no significant effect on FCHSD2 recruitment to CCPs (Figure S3B), suggesting that phosphoinositides are not primarily responsible for the recruitment of FCHSD2 to CCPs but rather modulate its function after recruitment.

To investigate which FCHSD2 domains are required for its recruitment to CCPs, we expressed a series of truncation constructs in cells and evaluated their colocalization with clathrin punctae. Only constructs that included the SH3-2 (FCHSD2 full length and F2B12) localized to CCPs (Figure 2B). Expression of the SH3-2 alone also formed punctae that colocalized with clathrin, albeit to a lesser extent when compared to constructs that also contained the F-BAR domain (Figure 2B). In addition, expression of these truncation constructs revealed that, similarly to what has been described for Nwk (Kelley et al., 2015), both SH3 domains cooperate to autoinhibit FCHSD2. This autoinhibition avoids promiscuous membrane binding (Figure S3C, as judged by formation of membrane protrusions and membrane binding in cells) and is predicted to be relieved when the SH3 domains are occupied by their cognate binding proteins. Thus, our data show that FCHSD2 is a bona fide CME component whose recruitment to CCPs is dependent on its second SH3 domain.

**Figure 3:**
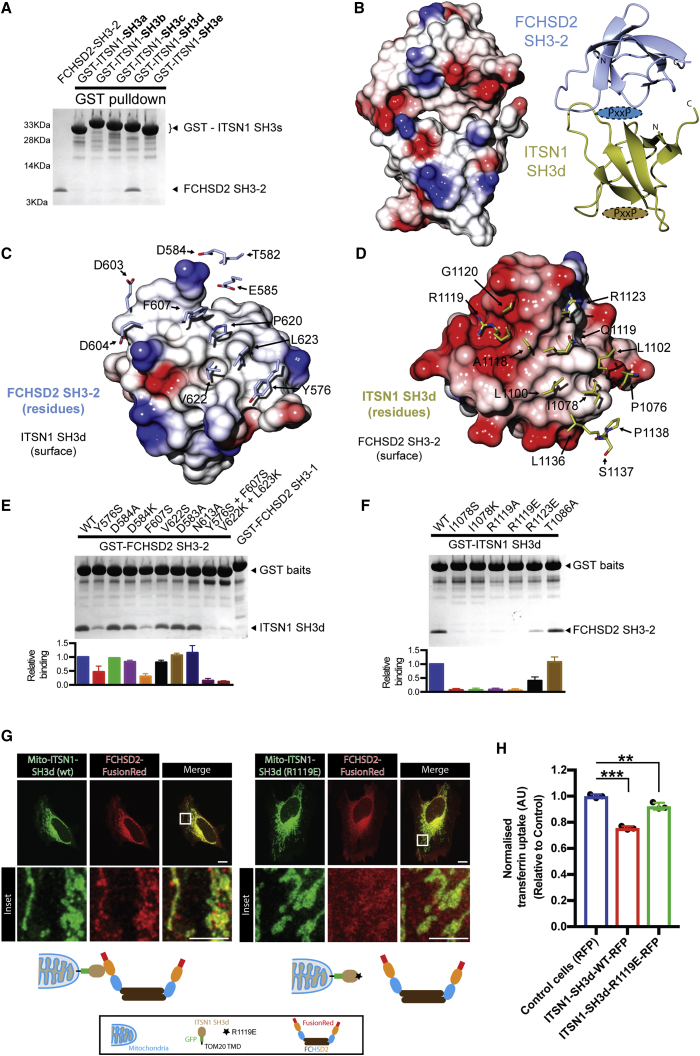
FCHSD2 Binds to Intersectin via an SH3-SH3 Interaction (A) Coomassie stained gel of GST pull-down experiments using individual ITSN1 SH3 domains as baits to bind to FCHSD2 SH3-2. (B) Overview of the co-crystal structure of the complex FCHSD2 SH3-2 and ITSN1-SH3d. Electrostatic surface (left) and backbone trace (right) of the complex. FCHSD2 SH3-2 is on top (in blue) and ITSN1-SH3d is on the bottom (in gold). Ovals with PxxP represent canonical proline-rich peptide binding surfaces for FCHSD2 SH3-2 (in blue) and ITSN1 SH3d (in gold). (C and D) Interaction surface of the FCHSD2 SH3-2/ITSN1-SH3d complex. Only the residues within contact distance (<4 Å) are shown for FCHSD2 SH3-2 (C, in blue) or ITSN1 SH3d (D, in gold). (E and F) Top: Coomassie stained gels for pull-down experiments using individual FCHSD2 SH3-2 (E) or ITSN1 SH3d (F) mutants of the interaction surface (and controls). Bottom: Quantification of the interaction of FCHSD2 SH3-2 mutants with ITSN1 SH3d (E) and ITSN1 SH3d mutants with FCHSD2 SH3-2 (F) (n = 3 experiments, mean ± SD). (G) Top: Confocal slice of HeLa cells transfected with FCHSD2-FusionRed plus mitochondrially targeted wild-type or interface mutant of ITSN1 SH3d. Bottom: Schematic explanation of the experiment. TOM20 TMD, TOM20 transmembrane domain. Scale bars, 10 μm in overviews, 5 μm in insets. (H) Transferrin uptake assay by flow cytometry comparing cells expressing RFP or RFP-ITSN1 SH3d (wild-type [WT] or interface mutant). Measurements were made from cells with high RFP signal. Results were normalized by the amount of surface transferrin receptor for each condition and against uptake for the RFP transfected cells. (n = 3 experiments with >5,000 gated cells, mean ± SD). ^∗∗∗^p > 0.001, ^∗∗^p > 0.01, one-way ANOVA with Tukey’s post hoc analysis. See also Figure S4 and Table S1.

**Figure S4:**
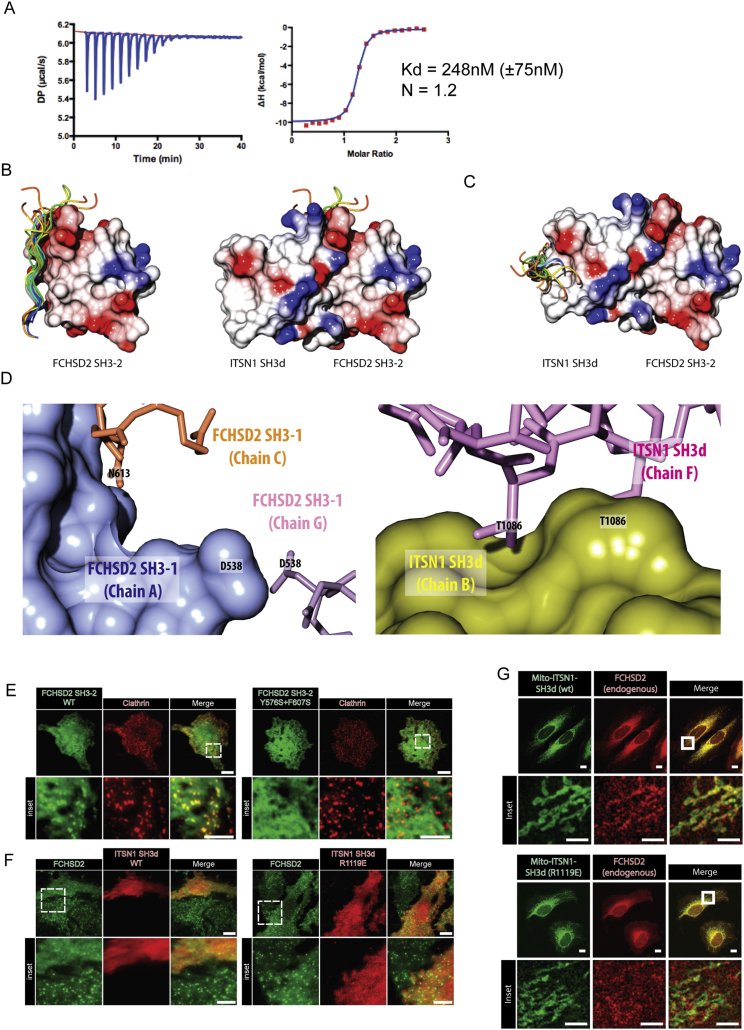
FCHSD2 Binds to Intersectin via an SH3-SH3 Interaction, Related to Figure 3 (A) Binding affinity for the FCHSD2 SH3-2 / ITSN1 SH3d interaction measured by isothermal titration calorimetry (ITC). (B) To highlight the canonical proline-binding interface of FCHSD2 SH3-2, it was aligned with 17 available structures of SH3 domains and their peptides (Structures used: 1N5z, 1sem, 1cka, 1abo, 1bbz, 1n5z, 1uj0, 1w70, 1ywo, 2df6, 2d1x, 2j71, 2o9v, 2vkn, 2v1r, 3u23, 4f14). Left: The structural alignment reveals the interface where proline-rich peptides (shown as ribbons) bind on FCHSD2 SH3-2 (shown as surface). Right: The ITSN1 SH3d (also shown as surface) binds to the canonical proline-binding interface of FCHSD2. (C) The ITSN1 SH3d was aligned to the same 17 structures as above. The structural alignment reveals that the interaction to FCHSD2 SH3-2 does not involve the proline-binding interface of ITSN1 SH3d. (D) Structural localization of residues used as negative control mutants. Localization of residues D538 and N613 in FCHSD2 SH3-1 (left) and T1086 in ITSN1 SH3d (right) forming homotypical crystal contacts. One of the chains is represented as surface to facilitate visualization. (E) TIRF images of HeLa cells transfected with mCherry-ClathrinLC plus wild-type or interface mutant of GFP-FCHSD2 SH3-2 (Y576S+F607S). The interface mutant does colocalize with clathrin punctae. (F) TIRF images of FCHSD2-Venus stable cells transfected with wild-type or interface mutant GFP-ITSN1 SH3d (R1119E). The wild-type construct act as dominant negative and displaces FCHSD2 from membrane punctae while the interface mutant does not. (G) Images of endogenous FCHSD2 staining in HeLa cells transfected with mitochondrially targeted wild-type or interface mutant of ITSN1 SH3d (R1119E). Scale bars = 10μm in overviews, 5μm in insets.

Given the key role of FCHSD2 SH3-2 in the localization of FCHSD2 to clathrin punctae, we tested if intersectin, a binding partner of FCHSD2 SH3-2 (Figure 1A), is required for FCHSD2 recruitment to CCPs. We performed knockdown for both intersectin isoforms (ITSN1/ITSN2) and quantified the colocalization between clathrin punctae and FCHSD2. Accordingly, cells silenced for ITSN1/2 showed a significant reduction of clathrin punctae colocalizing with FCHSD2 when compared to control cells (Figures 2C, 2D, and S3D). To further understand the relationship between intersectin and FCHSD2, we fine-mapped this interaction. Previous work mapped the interaction between the second SH3 of Nwk to a C-terminal fragment of dap160 containing multiple SH3s (O’Connor-Giles et al., 2008, Rodal et al., 2008). Taking this as a starting point, we tested the capacity of each of the five SH3 domains of ITSN1 (SH3a-e) to interact with FCHSD2 SH3-2 using GST pull downs. As shown in Figure 3A, the fourth SH3 domain of ITSN1 (SH3d) could pull down FCHSD2 SH3-2. Isothermal titration calorimetry (ITC) revealed a 248 nM (±75 nM) affinity (Kd) with a ∼1:1 stoichiometry (Figure S4A). Thus, FCHSD2 is recruited to CCPs by intersectin via an SH3-SH3 interaction.

SH3 domains generally bind short proline-rich sequences rather than mediate domain-domain interactions (Kurochkina and Guha, 2013). To understand how this unusual SH3-SH3 interaction occurs, we solved the crystal structure of the FCHSD2 SH3-2/ITSN1 SH3d complex at 3.4 Å (Table S1). The structure revealed tandem binding between the SH3 domains with the canonical proline-binding interface of FCHSD2 SH3-2 interacting with the surface opposite to the proline-binding interface of ITSN1 SH3d (Figures 3B, S4B, and S4C). The domains form an extensive contact surface composed largely of hydrophobic residues and a few charge interactions (Figures 3C and 3D). To confirm the SH3-SH3 contacts as the bona fide biological interface, we performed GST pull downs using a series of mutants for both SH3 domains. Double mutants on FCHSD2 SH3-2 (Y576S+F607S or V622K+L623K) were required to abolish the interaction to ITSN1 SH3d (Figure 3E). On the other hand, single residue mutations on ITSN1 SH3d (I1078S, I1078K, R1119A, or R1119E) could abolish the interaction with FCHSD2 SH3-2 (Figure 3F). Additionally, mutation of residues located on crystal contacts (FCHSD2 SH3-2 N613 and D583; ITSN1 SH3d T1086) did not show any effect on binding (Figures 3E, 3F, and S4D).

Next, we set out to validate in cells the biological significance of the FCHSD2 SH3-2/ITSN1 SH3d structure. In contrast to its wild-type counterpart, expression of the FCHSD2 SH3-2 interface mutant (Y576S+F607S) failed to localize to clathrin punctae (Figure S4E). On the other hand, expression of wild-type ITSN1 SH3d, but not the interface mutant counterpart R1119E, resulted in a substantial reduction of FCHSD2 punctae (Figure S4F). To further confirm the role of the FCHSD2 SH3-2/ITSN1 SH3d crystal interface on the correct localization of FCHSD2, we used the transmembrane domain of the mitochondrial translocase complex subunit TOM20 to mistarget the ITSN1 SH3d to the mitochondria. In agreement with the interface from the crystal structure, wild-type ITSN1 SH3d could efficiently recruit FCHSD2 to the mitochondria while the interface mutant ITSN1 SH3d R1119E could not (Figures 3G and S4G). Finally, we tested the functional significance of the FCHSD2 SH3-2/ITSN1 SH3d interaction. Expression of wild-type ITSN1 SH3d acted as a dominant negative and could inhibit transferrin uptake while the interface mutant counterpart ITSN1 SH3d R1119E had a much smaller effect (Figure 3H). Altogether, our results establish FCHSD2 as a CME protein recruited by intersectin via an SH3-SH3 interaction, and the ITSN1 SH3d overexpression can be used to interfere with FCHSD2 function.

### FCHSD2 Is a Strong Activator of Actin Polymerization in the Presence of Membranes

**Figure S5:**
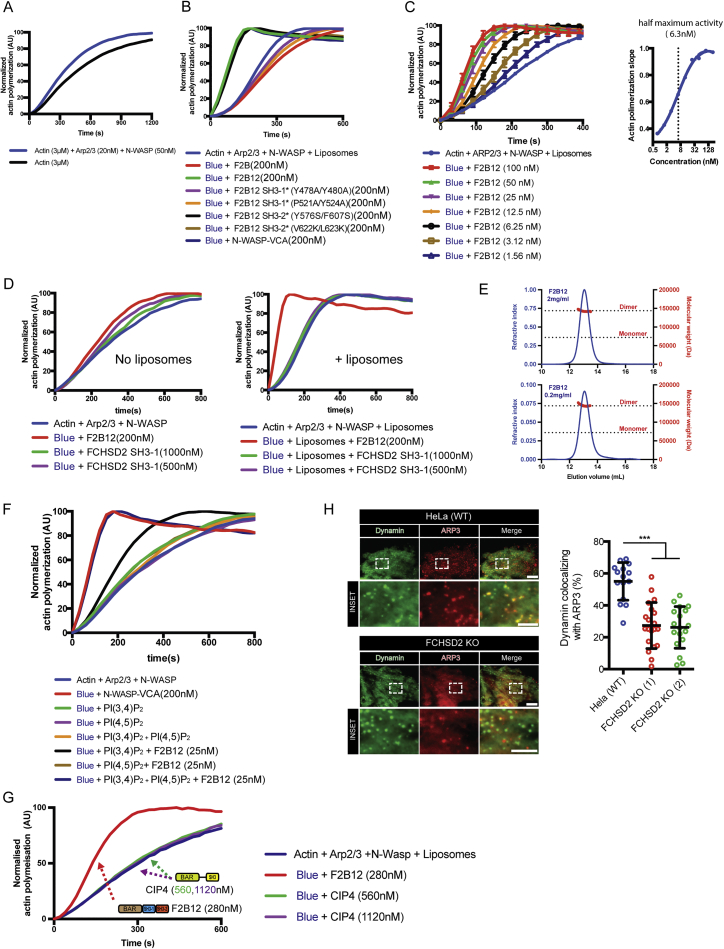
FCHSD2 Activates Actin Polymerization at CCPs, Related to Figure 4 (A) Comparison between polymerization reactions with actin alone and the minimal components Actin, ARP2/3 and N-WASP. (B) Full experiment as shown in Figure 4B including additional controls. (C) FCHSD2 strongly activates actin polymerization in the presence of liposomes. Left: Actin polymerization experiments using different FCHSD2 (F2B12 fragment) concentrations. Right: fitting of actin polymerization slopes versus F2B12 concentration. Vertical dashed line shows the half-maximum activity (6.3nM) (n = 3 experiments, mean ± SD). (D) The SH3-1 domain of FCHSD2 does not activate actin polymerization alone. Actin polymerization experiments using FCHSD2 SH3-1 at 500nM and 1μM in the absence (left) or presence of liposomes (right). The F2B12 fragment was used as a control. (E) SEC-MALS for FCHSD2 (F2B12 construct). Elution profiles and molecular weight determination for two concentrations of protein as indicated. Horizontal dotted lines indicate the predicted monomeric (71KDa) and dimeric (142KDa) masses of F2B12. Note that indicated protein concentrations refer to injected proteins. At elution volume, a 10-fold dilution is expected. (F) Full experiment as shown in Figure 4C including additional controls. (G) Actin polymerization experiment showing that CIP4 cannot activate actin polymerization without cdc42. (H) Effect of FCHSD2 knockout on ARP3 recruitment to CCPs. Images are time projections (20 s) of GFP-Dynamin and mCherry-ARP3. Right: Quantification of the percentage of Dynamin punctae colocalizing with ARP3 in control and FCHSD2 KO cells. (n = 15, 20, 18 cells for control, FCHSD2 KO(1) and FCHSD2 KO(2) respectively). Scale bars = 10μm in overviews, 5μm in insets. Data is displayed as mean ± SD. ^∗∗∗^p > 0.001, One-way ANOVA with Tukey’s post hoc analysis.

Another important feature of FCHSD2 is its relationship with the actin cytoskeleton via interaction of its first SH3 domain with the ARP2/3 complex activator N-WASP (O’Connor-Giles et al., 2008, Rodal et al., 2008) (Figure 1A). Activation of N-WASP via interaction with SH3 domains has been described for many proteins, including Nwk (Padrick and Rosen, 2010, Rodal et al., 2008). To test the capacity of FCHSD2 to activate N-WASP, we used a well-established *in vitro* assay (Doolittle et al., 2013) in which the kinetics of actin polymerization can be followed by monitoring the increase in the fluorescence of pyrene-labeled actin. For all reactions, we used a minimal set of components (actin, Arp2/3, and full-length N-WASP) where we could test the effect of various purified FCHSD2 fragments. Without any activator, this minimal set of components represents a baseline of actin polymerization where N-WASP is largely inactive and the majority of the signal comes from spontaneous actin nucleation (Figure S5A).

**Figure 4:**
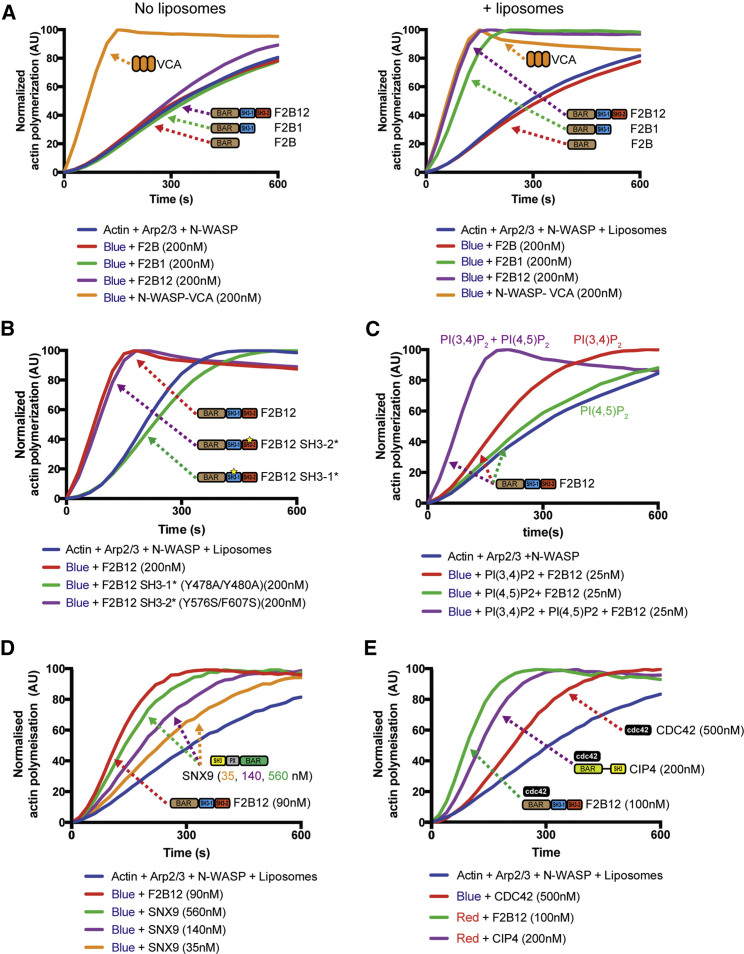
FCHSD2 Activates Actin Polymerization at CCPs All actin polymerization reactions were performed using 3 μM actin, 25 nM ARP2/3, and 50 nM N-WASP. Liposomes were used at 12.5 μM. The nomenclature for the fragments used is the same as in Figure 2B. (A) Actin polymerization experiments using different FCHSD2 truncations in the absence (left) and presence (right) of liposomes (Folch lipids, extruded with 800 nm filters). (B) Actin polymerization experiments using mutants disrupting the proline-binding pocket of SH3-1 and SH3-2. Full experiment including additional controls and one extra set of mutants is shown in Figure S5B. (C) Actin polymerization experiments using liposomes with different phosphatidylinositol composition. The full experiment including additional controls is shown in Figure S5F. (D and E) Actin polymerization experiments comparing the activity of FCHSD2 with SNX9 (D) and CIP4 in the presence of cdc42 (E). See also Figure S5.

In reactions containing only the minimal components, none of the tested FCHSD2 fragments showed a significant increase of actin polymerization when compared to the control reactions (Figure 4A). In contrast, when liposomes where added to the reaction, FCHSD2 fragments containing both the BAR domain and the SH3-1 (F2B1 and F2B12) activated actin polymerization to the same level as the positive control N-WASP-VCA (the uninhibited fragment of N-WASP (Rohatgi et al., 1999) (Figure 4A). We further confirmed the requirement of the FCHSD2 SH3-1 domain for the activation of N-WASP by mutating the canonical proline-binding site of the SH3-1 domain. These mutations (Y478A/Y480A or P521A/Y524A) rendered F2B12 incapable of increasing actin polymerization while mutants for the proline-binding site of FCHSD2 SH3-2 (Y576S/F607S or V622K/L623K) still maintained full activation capacity (Figures 4B and S5B). Titration of FCHSD2 demonstrated that it is capable of activating actin polymerization even at substoichiometric concentrations (half-maximal activity ∼6.3 nM in reactions containing 50 nM N-WASP) (Figure S5C). FCHSD2 SH3-1 alone could not significantly increase actin polymerization even at high concentrations (e.g., 1 μM) (Figure S5D), suggesting an active role of the interaction of FCHSD2 with the membrane on FCHSD2-dependent activation of N-WASP.

There are two levels of N-WASP regulation: one level is where the intramolecular autoinhibition of N-WASP is released by the action of allosteric activators such as PI(4,5)P_2_ and cdc42 (Rohatgi et al., 1999) and another level where oligomerization of N-WASP by multivalent interactors greatly increases its affinity for ARP2/3 complex (Padrick et al., 2008). The requirement of liposomes for efficient activation of N-WASP by FCHSD2 together with the fact that FCHSD2 is a dimer (Figure S5E), and therefore can bind two N-WASP molecules, suggests that FCHSD2 may act on these two regulatory levels of N-WASP activation. To test this possibility we used conditions that allowed us to differentiate the contribution of FCHSD2 on each of these levels. As PI(3,4)P_2_ is not an allosteric activator of N-WASP (Ma et al., 1998), we could test the effect of FCHSD2-mediated N-WASP oligomerization on membranes (using liposomes containing PI(3,4)P_2_) and compare it to conditions where oligomerization and recruitment to an allosteric activator would occur (using liposomes containing PI(3,4)P_2_ and PI(4,5)P_2_). As shown in Figure 4C, reactions with liposomes containing both PI(3,4)P_2_ and PI(4,5)P_2_ lipids showed maximum actin polymerization while reactions with PI(3,4)P_2_ liposomes showed an intermediate level of activation (Figures 4C and S5F). On the other hand, reactions containing PI(4,5)P_2_ liposomes showed no increase in actin polymerization (Figures 4C and S5F). These results show a contribution of both oligomerization and allosteric activation in FCHSD2-mediated N-WASP activation.

Our results in cells revealed FCHSD2 as a major activator of ARP2/3-dependent actin polymerization during CME (Figure 1H). To test this result *in vitro* we compared the actin polymerization activity of FCHSD2 with that of SNX9 and CIP4. These two F-BAR domain proteins are proposed to activate N-WASP during CME via direct activation of N-WASP (SNX9) (Yarar et al., 2007) or indirectly via cdc42 (CIP4) (Fricke et al., 2009). As shown in Figure 4D, FCHSD2 displayed much higher actin activation activity than SNX9 with 90nM FCHSD2 still showing higher activation capacity than 560 nM SNX9. As expected, in the absence of cdc42, CIP4 failed to activate actin polymerization (Figure S5G). In the presence of GTPγS-loaded cdc42, CIP4 showed strong actin activation activity, however, still to a lower degree than FCHSD2 (Figure 4E).

Next, we decided to test the connection between both of the features of FCHSD2: recruitment to CCPs and activation of actin polymerization. As N-WASP activation leads to the recruitment of the ARP2/3 complex to an existing actin filament, we used the accumulation of ARP3 at dynamin spots as a reporter for N-WASP activation. Confirming that FCHSD2 activates N-WASP at CCPs, FCHSD2 KO cells showed a significant reduction of ARP3 punctae colocalizing with dynamin in a live cell imaging experiment (Figure S5H). Taken together, these results show that FCHSD2 is a major activator of actin polymerization via N-WASP at CME sites.

### FCHSD2 Localizes to the Planar Membrane around CCPs

**Figure 5:**
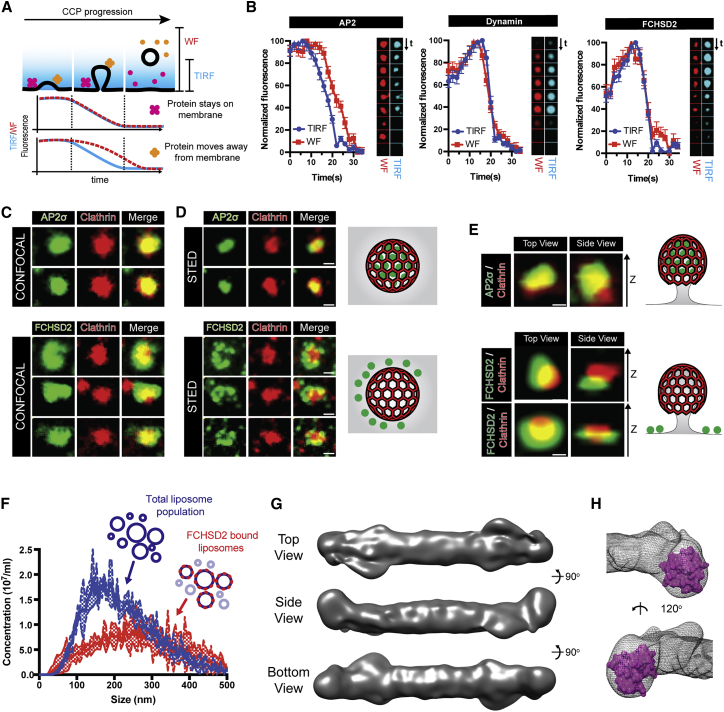
FCHSD2 Localizes to the Plasma Membrane Side of CCPs (A) Sequential widefield (WF) and TIRF imaging of endocytic proteins allows the distinction between proteins that stay on the membrane and proteins that move away from the membrane. (B) Results for AP2, dynamin, and FCHSD2 using the experimental paradigm explained in (A). Image series of representative events generated from videos at 0.5 Hz. (n = 54, 51, and 53 events for AP2, dynamin, and FCHSD2 respectively, mean ± SEM). (C–E) Comparative localization of AP2 and FCHSD2 with CCPs by confocal (C), STED (D), and 3D STED (E) microscopy. Stable HeLa cells for AP2σ2-GFP and FCHSD2-Venus cells were stained with anti-GFP and anti-Clathrin antibodies. Cartoon representations of the views are shown on the right hand side of super resolution images. Scale bars, 0.25 μm. (F) Curvature preference of FCHSD2 BAR by nanoparticle tracking analysis (NTA). Graph showing the size distribution of the total liposome population and the FCHSD2 BAR-sfGFP bound subpopulation. Total population distribution is measured by tracking particles diffracting light while FCHSD2 bound population is measured by tracking particles emitting light from GFP excitation. FCHSD2 BAR-sfGFP added at 1 nM (n = 3 experiments, mean ± SD). (G and H) Single particle cryoEM of F2B1 (FCHSD2 F-BAR+SH3-1). (G) 3D reconstruction from 30,207 particles. (H) The densities at the tip of the 3D map are compatible with an SH3 domain. In magenta is a surface representation of the FCHSD2 SH3-1 NMR structure (PDB: 2DL5). See also Figure S6.

The actin cytoskeleton forms a distinctive structure at CME sites (Collins et al., 2011). To understand how FCHSD2 contributes to the formation of this structure, we examined where on CCPs FCHSD2 was localized. We started by probing the relative Z-displacement of FCHSD2 during CCP lifetime. For this, we compared the fluorescence profiles of FCHSD2, AP2 (AP2σ2), and dynamin1 captured sequentially in total internal reflection fluorescence (TIRF) and widefield (WF). Using this imaging paradigm, proteins located in budding clathrin vesicles lose their signal on the TIRF channel (as the evanescent field is only ∼100 nm in depth) while maintaining it for a few more seconds on the WF channel. In contrast, if a protein stays on the plasma membrane during the whole lifetime of the CCP, the fluorescence decays at the same time for both TIRF and WF channels (Figure 5A). As expected for their established roles in CME, AP2 profiles showed a delayed fluorescence decay on the WF channel (i.e., AP2 buds off with the clathrin-coated vesicle), while dynamin fluorescence decayed at the same time on both channels (i.e., dynamin stays on the plasma membrane) (Figure 5B). FCHSD2 signal decayed at the same time in both TIRF and WF channels (Figure 5B), suggesting FCHSD2 is not located on or around the clathrin cage, but rather stays close to the plasma membrane during CME.

**Figure S6:**
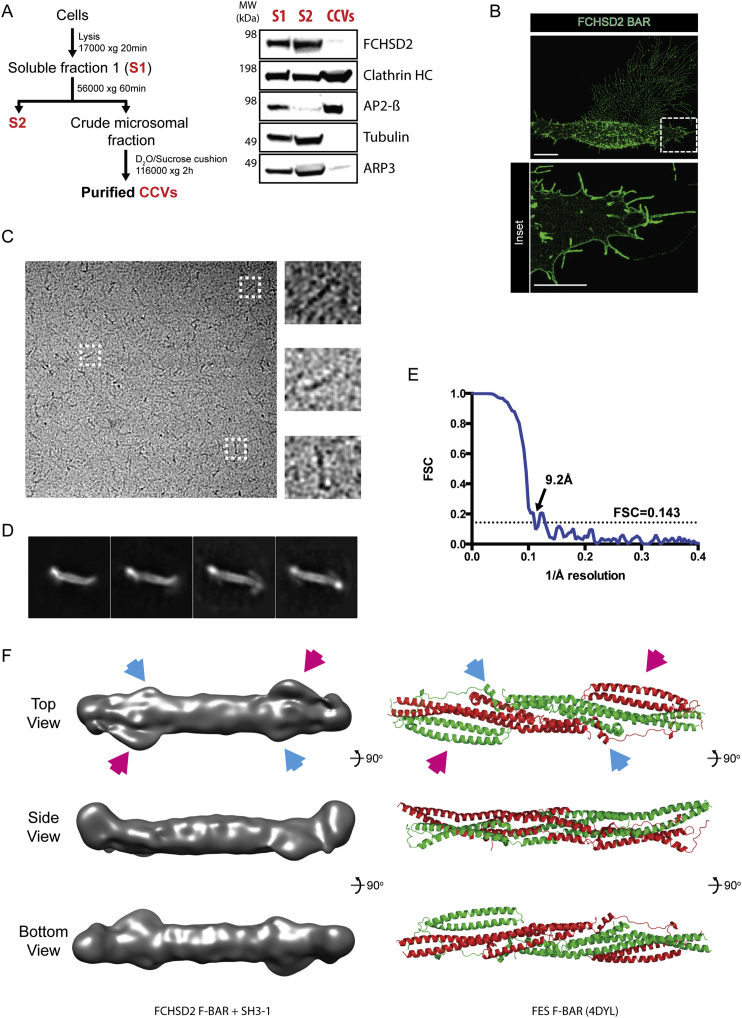
The FCHSD2 F-BAR Is Flat, Related to Figure 5 (A) Left: Simplified CCV purification protocol. Fractions in red were used for western blot. Right: western blot from CCV purification. 20μg of each fraction per lane. Note the enrichment of typical CCV markers (Clathrin and AP2 beta) in purified vesicles and the deenrichment for FCHSD2, tubulin and ARP3. (B) Super resolution Structured Illumination Microscopy (SIM) of overexpressed GFP tagged FCHSD2 F-BAR showing plasma membrane coating and the formation of cellular protrusions. Scale bars = 10μm in overview, 5μm in inset. (C) Representative electron micrograph and three examples of F2B1 particles on the right. (D) Selected 2D averages used for 3D reconstruction. (E) Estimation of the average resolution of the cryo-EM 3D reconstruction on the basis of the gold standard FSC criteria of 0.143. (F) Comparison between our F2B1 3D model (left) and the Fes F-BAR structure (PDB: 4DYL). Arrows point to similar features between the FCHSD2 F-BAR and the FES F-BAR.

To further address the localization of FCHSD2 on CCPs, we imaged FCHSD2 and AP2 with stimulated emission depletion (STED) super-resolution microscopy. While AP2 showed a complete colocalization with clathrin, FCHSD2 formed multiple punctae arranged in a semi-circle (sometimes a circle) around CCPs (Figures 5C and 5D). To further confirm the position of FCHSD2 on CCPs, we used 3D STED, which allows increased resolution imaging at the Z plane (albeit at the expense of lower resolution at XY). The orthogonal (XZ) view of FCHSD2 and clathrin by 3D STED confirmed that FCHSD2 is localized around and at the plasma membrane side of CCPs (Figure 5E). In agreement with FCHSD2 being localized out of the boundaries of the budding CCP, FCHSD2 was not present in purified CCVs (Figure S6A).

F-BAR domains are elongated, slightly curved, dimeric membrane-binding domains. The intrinsic shape of these domains determines the curvature of the membrane they are bound to (Qualmann et al., 2011). As such, expression of most F-BAR domains generates inward tubules in cells (Boucrot et al., 2012, Frost et al., 2008). In contrast, expression of FCHSD2 (and Nwk) F-BAR domain generates large numbers of filopodia-like cellular protrusions (Figure S6B) (Kelley et al., 2015). Interestingly, we observed that overexpressed FCHSD1/2 F-BAR domains coat the whole cellular membrane and not only the protrusions it induces (Figure S6B, inset). The data we present here shows that FCHSD2 is concentrated on a region of the membrane that is primarily flat, suggesting that FCHSD2 F-BAR is either distinctly shaped or has a distinct mode of binding to membranes. To investigate this atypical behavior, we started by checking the curvature preference of FCHSD2 *in vitro*. For that, we adapted the nanoparticle tracking analysis (NTA) system (Dragovic et al., 2011) to determine curvature preferences of protein domains to liposomes using low protein concentrations and avoiding curvature generation artifacts (A.C., L.A.-S., and H.T.M., unpublished data). This method is able to distinguish to which liposome sizes a particular protein binds within a population of heterogeneously sized liposomes. In agreement with its cellular localization, NTA shows that FCHSD2 binds preferentially to larger liposomes (Figure 5F).

To understand the molecular basis of FCHSD2 curvature preference, we used single particle CryoEM to determine the structure of the FCHSD2 F-BAR domain + SH3-1 domain (F2B1) (Figures S6C and S6D). We obtained a 9.2 Å reconstruction (Figure S6E) that reveals a largely flat F-BAR with densities on its tips compatible with the SH3-1 domains (Figures 5G and 5H). Furthermore, our FCHSD2 F-BAR map shows similarities with the crystal structure of the FES F-BAR (PDB: 4DYL, no associated publication), which is also a flat F-BAR domain (Figure S6F). Altogether, our results show that FCHSD2 stays on the surrounding plasma membrane during CME and binds to membranes via its atypical, flat F-BAR domain.

## Discussion

### CME and Its Multiple Actin Regulators

The functional connection between the actin cytoskeleton and CME has been a matter of controversy for more than a decade. While this relationship is well established in yeast (Merrifield and Kaksonen, 2014), its importance in mammalian systems seems to be context- or cell-dependent (Boulant et al., 2011, Fujimoto et al., 2000). Nonetheless, a growing body of evidence supports the idea that actin is an intrinsic component of mammalian CCPs (Ferguson et al., 2009, Grassart et al., 2014) and modulates the effectiveness of CCP budding. Moreover, the intimate connection between CCPs and the actin cytoskeleton is further reinforced by a large number of CME proteins showing actin-related activities such as HIP1/HIP1R, the long isoforms of ITSN1/2 and notably, multiple F-BAR proteins (Daumke et al., 2014, McMahon and Boucrot, 2011, Qualmann et al., 2011). In which conditions these proteins are required, what their relative contribution is, and how they are organized to optimize the mechanical action of the actin cytoskeleton during CME remains unclear.

The unique position of FCHSD2 at the planar membrane region surrounding CCPs suggests a possible explanation for the apparent redundancy of actin regulators during CME. By coordinating the position and the timing of each actin regulator, cells would be able to change the strength and direction of actin mechanical forces during multiple stages of vesicle maturation. It is reasonable to assume that the multiple geometries acquired by the clathrin coat and the underlying budding membrane as CCPs mature would benefit from distinct force vectors. This possibility is supported by a recent work showing that, at least in yeast, different types of actin network are required during different stages of endocytosis (Picco et al., 2018). In addition to the distribution of force vectors during CME, the contribution of each individual actin regulator may also be tissue- and cargo-dependent or be involved in the transport of vesicles post scission. Future efforts to access the spatiotemporal organization of actin regulators in multiple cells during CME would be necessary to test these hypotheses.

### A Model for FCHSD2 Regulation and Function

In this study, we moved away from using actin polymerization inhibitors because of their toxic effects in cells. Using the inactivation of the ARP2/3 complex, we could obtain a consistent baseline for the contribution of branched actin polymerization during CME and determine that FCHSD2 is responsible for a large part of this contribution (Figure 1H). We cannot rule out that ARP3 KD can have indirect effects on CME by affecting, for example, membrane tension. Nonetheless, if these side effects occured, they would imply an even higher importance of FCHSD2 to the ARP2/3 contribution to CME. The relative importance of FCHSD2 to CME was further reinforced by the reduced recruitment of ARP3 to CCPs in FCHSD2 KO cells (Figure S5H), and a similar contribution measured by transferrin uptake. In addition, FCHSD2 activated actin polymerization via N-WASP *in vitro* more efficiently than the F-BAR proteins we tested (Figures 4 and S5).

The timing of FCHSD2 recruitment combined with the phenotypes of FCHSD2 KO cells (increased CCP lifetimes and the accumulation of U-shaped pits) show that FCHSD2 has a kinetic role at the intermediate stage of CCP maturation, when the transition from a shallow invagination to a constricted pit ready for scission occurs. During this transition, the actin cytoskeleton at CCPs evolves into a cone-shaped, densely branched network with its origin at the flat region around CCPs (Collins et al., 2011). The position and function of FCHSD2 strongly suggests that this protein is involved in the generation of this actin structure.

Our data suggest that recruitment and activation of FCHSD2 are stepwise events. While the presence of FCHSD2 in CCPs is dependent exclusively on intersectin, FCHSD2 activation seems to be dependent on the phosphoinositide PI(3,4)P_2_. Further supporting a stepwise cascade of events, both SH3 domains participate in FCHSD2 autoinhibition (Figure S3C). We believe that this uncoupling allows cells to spatiotemporally control FCHSD2 activation. A recent work elegantly showed that intersectin migrates to the edge of the clathrin coat as CCPs mature (Sochacki et al., 2017). Therefore, we hypothesize that FCHSD2 is recruited by intersectin and is kept in an inhibited/low activity state until it reaches the edge of the CCP, where the accumulation of PI(3,4)P_2_ recruits FCHSD2 to the flat membrane triggering actin polymerization.

**Figure 6:**
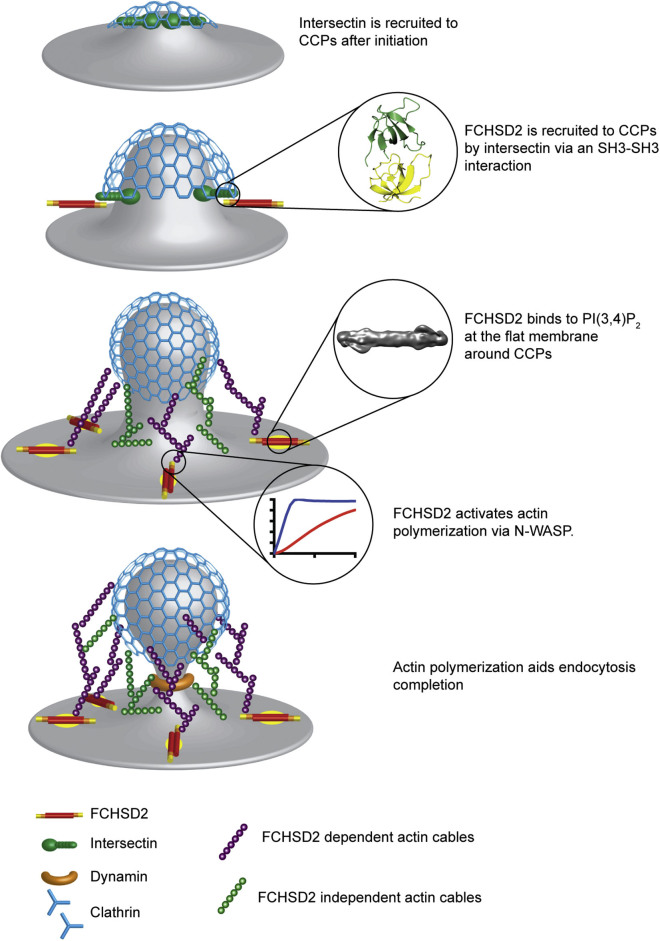
Model for FCHSD2 Function in CME See text for details.

Taking all this information into account, we propose the following model for FCHSD2 function (Figure 6). After CME initiation, FCHSD2 is recruited to CCPs by intersectin via an SH3-SH3 interaction and intersectin-FCHSD2 complexes accumulate at the edge of CCPs. The flat FCHSD2 F-BAR domain and presence of the late phosphoinositide PI(3,4)P_2_ drives the binding of FCHSD2 to the planar region apposed to CCPs and allows it to activate actin polymerization. The FCHSD2-dependent actin structure around CCPs helps the efficient invagination of the maturing pit. Finally, other CME players (both actin and actin-independent) take over and ensure the completion of the process.

### The Rise of Non-canonical F-BARs

The BAR superfamily is characterized by the presence of an elongated, dimeric membrane-binding domain (Qualmann et al., 2011). The superfamily is further subdivided into N-BAR, F-BAR, and I-BAR families. While this division is primarily based on the homology between these domains, the initial crystal structures described suggested that this classification also implied a shape of the molecule and by consequence its curvature preference (Frost et al., 2008, Henne et al., 2007, Qualmann et al., 2011, Rao and Haucke, 2011). While for N-BAR and I-BAR proteins this correlation still holds true, the F-BAR family has shown to be far more flexible regarding its curvature preference. The first non-canonical F-BAR characterized were the SRGAP proteins that were shown to generate large filopodia when expressed in cells (Coutinho-Budd et al., 2012). The crystal structure of SRGAP F-BAR domain revealed an inverted F-BAR, with its lipid binding sites situated in the convex rather than the concave surface (Sporny et al., 2017). Similarly, the structure of the FES F-BAR domain revealed a flat BAR domain (PDB: 4DYL, no associated publication). The CryoEM map we present here adds to the list of unusual F-BARs and supports the idea of the F-BAR domain as a module used by evolution to recognize multiple curvatures.

Why would cells need a BAR domain to recognize a flat membrane? The FCHSD2 F-BAR is a large domain (467 aa), and one could argue that a small globular domain, such as a PH domain, could do the same job. We reason that a flat BAR domain with multiple binding sites scattered over its length provides a structural framework to ensure that FCHSD2 binds only to the planar membrane around the CCPs rather than competing for other phosphoinositol lipids on the curved regions of the budding membrane. Small globular membrane binding domains are exquisite at recognizing specific lipids. However, with a footprint of only a few lipid headgroups, small domains on their own are possibly unable to read the long-range differences that distinguish a flat membrane from a curved one.

### Beyond CME

Besides its CME role, intersectin has been described to participate in a series of other processes including other endocytic routes, exocytosis, and viral entry (Herrero-Garcia and O’Bryan, 2017). The intimate connection between FCHSD2 and intersectin and the affinity of FCHSD2 to the pivotal signaling phosphoinositol PI(3,4,5)P_2_ opens the possibility that the combined actin remodeling capacity of these proteins are used in processes other than CME.

### Conclusion

In conclusion, our work places FCHSD2 as a pivotal player regulating actin polymerization during CME. The unique position of FCHSD2 on the planar membrane around CCPs is, to our knowledge, the first description of a CME-specific mammalian protein localized on a region other than the clathrin coat or the neck of the CCPs. The mechanism we describe for FCHSD2 illustrates how cells link in space and time the formation of CCPs and the reorganization of the cortical actin cytoskeleton during mammalian endocytosis. These findings also provide a mechanistic framework that may be applicable to other membrane bending events involving the actin cytoskeleton.

## STAR★Methods

### Key Resources Table

**Table:** 

REAGENT or RESOURCE	SOURCE	IDENTIFIER
**Antibodies**		

Rabbit polyclonal anti-FCHSD2 (F2B1)	This paper	N/A
Rabbit polyclonal anti-FCHSD2 (F2S2)	This paper	N/A
Rabbit polyclonal anti-ITSN1 (Clone S750)	A grift from Thomas Sudhof (Stanford, USA)	N/A
Rabbit polyclonal anti-GFP	Abcam	Cat# Ab290
Mouse monoclonal anti-integrinß1 (clone 12G10)	Santa cruz biotechnology	Cat# Sc-59827
Anti-ARP3 (Clone FMS338)	Sigma-Aldrich	Cat# A5979
Mouse monoclonal anti-PI3KC2α (clone 17)	BD biosciences	Cat# 611046
Rabbit monoclonal anti-Nwasp	Abcam	Cat# Ab126626
Mouse monoclonal anti-CHC (clone x22)	Thermo Fisher scientific	Cat# MA1-065
Mouse monoclonal Anti transferrin receptor (clone H68.4)	Thermo Fisher scientific	Cat# 13-6800
Mouse monoclonal Anti-adaptin beta1,2 (clone 100/1)	Thermo Fisher scientific	Cat# MA1-25065

**Bacterial and Virus Strains**		

*E.coli* Rosetta (DE3)	Novagen	Cat# 70954

**Chemicals, Peptides, and Recombinant Proteins**		

Fibronectin	Sigma-Aldrich	Cat# F1141
Total Brain extract lipids (Folch)	Sigma-Aldrich	Cat# B1502
Total Brain extract lipids (Folch)	Avanti polar lipids	Cat# 131101P
2-Oleoyl-1-palmitoyl-*sn*-glycero-3-phosphocholine (PC)	Sigma-Aldrich	Cat# 42773
3-*sn*-Phosphatidylethanolamine (PE)	Sigma-Aldrich	Cat# P7693
1,2-Diacyl-*sn*-glycero-3-phospho-L-serine (PS)	Sigma-Aldrich	Cat# P6641
Cholesterol	Sigma-Aldrich	Cat# 47127
PI(3)P	Avanti polar lipids	Cat# 850150
PI(4)P	Avanti polar lipids	Cat# 850158
PI(5)P	Avanti polar lipids	Cat# 850152
PI(3,4)P2	Avanti polar lipids	Cat# 850183
PI(4,5)P2	Sigma-Aldrich	Cat# 840046
PI(3,4,5)P3	Avanti polar lipids	Cat# 850166
ARP2/3 complex	Cytoskeleton inc	Cat# RP01P
Pyrene labeled actin	Cytoskeleton inc	Cat# AP05
Transferrin Alexa fluor 488	Thermo Fisher scientific	Cat# T13342
In-Fusion HD cloning kit	Clontech	Cat# 638911
Gateway LR clonase	Thermo Fisher scientific	Cat# 11789100
Gateway BP clonase	Thermo Fisher scientific	Cat# 11791100
Lipofectamine LTX	Thermo Fisher scientific	Cat# A12621
Lipofectamine RNAiMax	Thermo Fisher scientific	Cat# 13778150

**Deposited Data**		

FCHSD2 SH3-2/ITSN1 SH3d crystal structure	This paper	PDB: 6GBU
FCHSD2 F-BAR + SH3-1 CryoEM map	This paper	EMDB: EMD-4371

**Experimental Models: Cell Lines**		

HeLa cells	ATCC	Cat# CCL2
HeLa FCHSD2 KO (clone 1)	This study	N/A
HeLa FCHSD2 KO (clone 2)	This study	N/A
Hek293T cells	ATCC	Cat# CRL-3216
Flp-in T-Rex HeLa cells	A gift from Dr. Stephen Taylor (U. Manchester, UK)	N/A
BSC1 cells stably expressing the adaptin σ2 subunit of the AP2 complex	Ehrlich et al., 2004	N/A
HeLa cells stably expressing the adaptin σ2 subunit of the AP2 complex	This study	N/A
Sf9 cells	ATCC	Cat# CRL-1711

**Oligonucleotides**		

ITSN1 siRNA (esiRNA)	Sigma-Aldrich	Cat# EHU102031
ITSN2 siRNA (esiRNA)	Sigma-Aldrich	Cat# EHU064971
ARP3 siRNA (esiRNA)	Sigma-Aldrich	Cat# EHU107121
PI3KC2α siRNA pool	Origene	Cat# SR303516

**Recombinant DNA**		

pcDNA5/FRT/TO/Venus Gateway destination vector	A gift from Jonathon Pines (Gurdon institute, Cambridge, UK)	N/A
pCDNA5/FRT/TO-FCHSD2-Venus	This study	N/A
FCHSD2 shRNAs	Sigma-Aldrich	TRCN0000147537, TRCN0000147035, TRCN0000148919
pFUW-AP2σ2-GFP	Ehrlich et al., 2004	N/A
pSpCas9(BB)-2A-GFP (PX458)	Ran et al., 2013	Adgene (Cat# 48138)
PX458-FCHSD2_gRNA2 (ACTTCAAGCCAAACATCAAG)	This study	N/A
pLenti6/V5-Dest	Thermo Fisher scientific	Cat# V49610
pLenti6-FCHSD2	This study	N/A
pOPINS (N-terminal His-SUMO tag)	Oxford protein production facility	N/A
pOPINS-FCHSD2 FCHSD2 BAR (F2B: aa1-468)	This study	N/A
pOPINS-FCHSD2 BAR + SH3-1 (F2B1: aa1-530)	This study	N/A
pOPINS-FCHSD2 BAR + SH3-1 + SH3-2 (F2B12: aa1-629)	This study	N/A
pOPINS-FCHSD2 SH3-1 (aa469-530) (wt and mutants: Y478A+Y480A; P521A/Y524A)	This study	N/A
pOPINS-FCHSD2 SH3-2 (aa568-629) (wt and mutants: Y576S+F607S; V622K+L623K)	This study	N/A
p7x- FCHSD2 BAR-sfGFP (C-terminal sfGFP-6xHis)	This study	N/A
pCI-FCHSD2 - EGFP	This study	N/A
pCI-FCHSD2 - FusionRed	This study	N/A
pCI-FCHSD2 FCHSD2 BAR - EGFP (F2B-GFP)	This study	N/A
pCI-FCHSD2 BAR + SH3-1 - EGFP (F2B1-GFP)	This study	N/A
pCI-FCHSD2 BAR + SH3-1 + SH3-2 - EGFP (F2B12-GFP)	This study	N/A
pCI-EGFP-FCHSD2 SH3-1 (GFP-F2S1)	This study	N/A
pCI-EGFP-FCHSD2 SH3-2 (GFP-F2S2) (wt and Y576S+F607S)	This study	N/A
pCI-RFP-ITSN1 SH3d (wt and R1119E)	This study	N/A
pCI MitoGFP-ITSN1 SH3d (wt and R1119E)	This study	N/A
pGEX6P2- FCHSD2 SH3-1 (aa469-530) (N-terminal GST)	This study	N/A
pGEX6P2- FCHSD2 SH3-2 (aa568-629) (N-terminal GST) (WT and mutants: Y576S; D584A; D584K; F607S; V622S; D583A; N613A; Y576S+F607S; V622K+L623K)	This study	N/A
pGEX6P2- FCHSD1 SH3-1 (aa466-527) (N-terminal GST)	This study	N/A
pGEX6P2- FCHSD1 SH3-2 (aa544-607) (N-terminal GST)	This study	N/A
pGEX6P2- ITSN1 SH3a (aa740-806) (N-terminal GST)	This study	N/A
pGEX6P2- ITSN1 SH3b (aa913-971) (N-terminal GST)	This study	N/A
pGEX6P2- ITSN1 SH3c (aa10021060) (N-terminal GST)	This study	N/A
pGEX6P2- ITSN1 SH3d (aa1076-1138) (N-terminal GST) (WT and mutants:I1078S; I1078K; R1119A; R1123E; T1086A)	This study	N/A
pGEX6P2- ITSN1 SH3e (aa1155-1214) (N-terminal GST)	This study	N/A
pOPINS-ITSN1-SH3d (aa1076-1138)	This study	N/A
pOPINS-cdc42	This study	N/A
pOPINS-CIP4	This study	N/A
pOPINS-SNX9	This study	N/A
pOPINS-N-WASP VCA (aa385-501)	This study	N/A
pACEBAC1-N-WASP	This study	N/A
mCherry-ClathrinLC	A gift from Dr. Christien Merrifield (I2BC, Paris, France)	N/A
mCherry-ARP3	A gift from Dr. Christien Merrifield (I2BC, Paris, France)	N/A
FusionRed-ITSN1L	This study	N/A
FusionRed-Dynamin1	This study	N/A
Integrinβ3-BFP	This study	N/A

**Software and Algorithms**		

ImageJ (Fiji)	Schindelin et al., 2012	https://fiji.sc/
Nanosight NTA software version 3.1	Malvern	https://www.malvernpanalytical.com
MOSFLM	Battye et al., 2011	N/A
Scala	Evans, 2006	N/A
Phaser-MR	McCoy, 2007	N/A
Coot	Emsley et al., 2010	N/A
REFMAC	Murshudov et al., 2011	N/A
CCP4MG	McNicholas et al., 2011	N/A
Relion	Scheres, 2012	N/A
Chimera	Pettersen et al., 2004	N/A
Graphpad Prism 6.0	N/A	https://www.graphpad.com

### Contact for Reagent and Resource Sharing

Further information and requests for reagents should be directed to and will be fulfilled by the Lead Contact, Leonardo Almeida-Souza (lalmeida@mrc-lmb.cam.ac.uk).

### Experimental Model and Subject Details

#### Cell lines

HeLa (female), and BSC1 (unknown gender) cells were cultured in MEM (Thermo Fisher scientific) while HEK293T (female) were cultured in DMEM (Thermo Fisher scientific). In all cases media was supplemented with 10% fetal bovine serum (FBS) and 1 mM GlutaMAX-I (Thermo Fisher scientific).

HeLa Flp-in T-Rex (female) stables cells were selected and kept in MEM media supplemented with 10% fetal bovine serum (FBS) and 1 mM GlutaMAX-I (Thermo Fisher scientific) and 150 μg/ml hygromycin B (Thermo Fisher scientific) and 5 μg/ml blasticidin S (Thermo Fisher scientific).

For all microscopy experiments, HeLa cells were cultured in fibronectin-coated dishes (20 μg/ml, 2-4 h). We observed that the fibronectin coating reduces the amount of clathrin plaques in HeLa cells and allows the visualization of individual endocytic events. A more detailed explanation of this finding will be published elsewhere (L.A.-S. and H.T.M., unpublished data).

### Methods Details

#### FCHSD2 Antibodies

The rabbit polyclonal FCHSD2 antibodies used throughout this study were produced by David’s Biotechnologie (Germany) using recombinant F2B1 (used for immunoblots) or FCHSD2 SH3-2 (used for immunofluorescence). None of the FCHSD2 antibodies commercially available displayed a reduction of FCHSD2 in knockdown or knockout cells by western blot or immunofluorescence.

#### Constructs

All constructs for mammalian expression and GST fusion expression were made by Gateway recombination (Thermo Fisher scientific). cDNA reference sequences used are: FCHSD2 (human NM_014824), Fchsd1 (mouse NM_175684), ITSN1L (human, NM_003024), Dynamin1 (Bovine NM_001076820), Integrin β3 (human, NM_000212), CIP4 (human, NM_004240), SNX9 (Human, NM_016224), cdc42 (Human, NM_001791). For protein production in *E. coli* using 6xHis-SUMO tag, PCR fragments were cloned in pOPINS via In-Fusion recombination (Clontech). For N-WASP purification in baculovirus, the coding sequence of N-WASP (mouse NM_028459) was amplified using an oligo including an N-terminal 6xHis tag and cloned in pACEBAC1 via In-Fusion recombination (Clontech). FCHSD2 shRNA constructs were purchased from Sigma (TRCN0000147537, TRCN0000147035, TRCN0000148919). Boundaries for constructs used are as follows: FCHSD2 BAR (F2B: aa1-468); FCHSD2 BAR + SH3-1 (F2B1: aa1-530); FCHSD2 BAR + SH3-1 + SH3-2 (F2B12: aa1-629); FCHSD2 SH3-1 (aa469-530); FCHSD2 SH3-2 (aa568-629); ITSN1 SH3a (aa740-806); ITSN1 SH3b (aa913-971); ITSN1 SH3c (aa10021060); ITSN1 SH3d (aa1076-1138); ITSN1 SH3e (aa1155-1214); N-WASP VCA (aa385-501). Mito-ITSN1 constructs were generated by site directed mutagenesis by adding the 33 first aminoacids of TOM20 (MVGRNSAIAAGVCGALFIGYCIYFDRKRRSDPN) to the N terminus of the GFP-ITSN1 constructs. Constructs mCherry-ClathrinLC and mCherry-ARP3 were a gift from Dr. Christien Merrifield (Institut de Biologie Intégrative de la Cellule - I2BC, Paris, France). The AP2σ2-GFP construct was a gift from Dr. Tomas Kirchhausen (Harvard, Boston, USA). The pcDNA5/FRT/TO/Venus Gateway destination vector was a gift from Jonathon Pines (The Gurdon Institute, Cambridge, UK). pSpCas9(BB)-2A-GFP (PX458) was a gift from Feng Zhang (Addgene plasmid # 48138).

#### Generation of stable cell lines

FCHSD2-Venus HeLa Flp-in T-Rex cells were generated following the protocol for the generation of Flp-in expression cells (Thermo Fisher scientific) using full length FCHSD2 cloned into the destination vector pcDNA5/FRT/TO/Venus. Transgene expression was induced by addition of 1μg/ml tetracycline. HeLa AP2σ2-GFP stable cells were generated by transfecting the vector pFUW-AP2σ2-GFP followed by selection with G418 (500μg/ml) for two weeks. A low expressing clone was selected for experiments.

To generate HeLa FCHSD2 CRISPR/Cas9 knockouts, cells were transfected with 3 different gRNAs (AGCATCATGCAGCCGCCGCC, ACTTCAAGCCAAACATCAAG, CAGAAGAAGGCTGCTATTGA) cloned into pX458 (Ran et al., 2013). Individual clones were selected by limiting dilution and screed for FCHSD2 expression by western blot. The second gRNA yielded a few complete knockout clones. Two of them were selected for further experiments.

FCHSD2 knockdown cell lines were generated by lentiviral transduction of HeLa and BSC1 cells with shRNA constructs and selected with puromycin (2 μg/ml for HeLa, 10 μg/ml for BSC1). The FCHSD2 rescue cell line was generated by lentivirus transduction of the CRISPR KO cells with a virus encoding the full-length untagged FCHSD2 cloned in pLenti6/V5-Dest (Thermo Fisher scientific) and selected with blasticidin (10 μg/ml).

For siRNA knockdown, cells were transfected with Lipofectamine RNAiMax (Thermo Fisher scientific) following manufacturer instructions. Experiments were performed 72 h after transfection.

#### Cell preparation for microscopy

For TIRF and Spinning disk imaging, cells were seeded in 35 mm glass-bottom dishes (MatTek) and transfected after 24 h. Cells were imaged without fixation for single pictures and time-lapse videos. For STED microscopy, HeLa FCHSD2-Venus and HeLa AP2σ2-GFP cells were seeded in 13 mm glass coverslips, fixed with PFA (4%, 15 min, Ice), washed with PBS and stained with anti-GFP(1:100) and anti-CHC (1:50) overnight at 4°C, followed by PBS washes (5x 5 min) and a 2 h incubation with secondary antibodies at 1:100 dilution. After secondary incubation, coverslips were washed with PBS (5x 5 min) and mounted using ProLong diamond (Thermo Fisher scientific). Slides were left at room temperature for 24-48 h to cure the mountant before imaging.

#### Microscopy

TIRF images were acquired in a Nikon N-STORM microscope controlled by Nikon Elements software using a 100x objective. STED and confocal images were acquired in a Leica TCS SP8 X gated STED microscope controlled by Leica Application Suite X (LAS X) software equipped with a tunable pulsed white light laser for excitation and 592 nm and 660 nm depletion lasers using 100x or 63x objectives. SIM images were acquired in ZeissElyra S.1 and processed using the Zeiss acquisition software ZEN using a 63x objective. Spinning disk images were acquired using a Nikon TE2000 microscope equipped with a CSU-X1 spinning disk confocal head (UltraVIEW VoX, Perkin-Elmer) controlled by Volocity using a 63x objective.

#### Live cell imaging

For FCHSD2 dynamics with CME components, TIRF movies were taken at 1 frame/s using 100-300 ms exposure times. For CCP lifetime with BSC1 cells expressing AP2σ2-GFP, spinning disk movies were taken at 0.5 frame/s using 600 ms exposure times. For dynamic colocalization of dynamin and ARP2/3, TIRF movies were taken at 0.5 frame/s using ∼300 ms exposure times. For the dynamics of FCHSD2, clathrin and integrins, TIRF movies were taken at 3 frames/min using ∼300 ms exposure times.

#### Image analysis

All images were analyzed using ImageJ (Schindelin et al., 2012). In brief, analyses were done as follows:

##### CCP dynamics:

Dynamic events were identified by selecting brightest spots from a standard deviation projection using 50 frames in the middle of movies. For every selected event, maximum fluorescence for the reference channel (i.e., FCHSD2) was identified and the fluorescence profile for both channels of 50 frames on each side of the maximum fluorescence was selected. Traces were manually selected for events showing fluorescence profiles compatible with typical endocytic events. The use of a common reference point for all traces ensures the fluorescence profiles are aligned and allows direct comparison and averaging of all events. Curves were smoothed and normalized for display.

##### Colocalization:

Fluorescence maxima on both channels identifying clear spots were selected from images and converted into binary spots. The spots for the reference channel were expanded to 3x3 pixels and colocalizing spots were identified by image subtraction.

##### Widefield/TIRF fluorescence decay:

Events with clear signal on both widefield and TIRF channels were identified by visual inspection of movies and selected by drawing a 8x8 pixels box (0.1 μm/pixel) around them. Using the first frame where the TIRF signal disappears as reference, fluorescence profiles of 20 frames on each side of this reference point were selected. The use of a common reference point for all traces ensures the fluorescence profiles are aligned and allows direct comparison and averaging of events.

##### CCP Lifetime:

Dynamic events were identified by selecting brightest spots from a standard deviation projection using 20 frames in the middle of movies. A line connecting all dynamic events was traced to generate kymographs. CCP lifetimes were calculated by measuring the length of vertical traces on kymographs.

#### Wound healing assay

30000 cells were seeded in 8-16 replicates in ImageLock 96 well plates (Essen Biosciences). On the next day, cultures were scratched using a WoundMaker (Essen Biosciences) and imaged for 48 hours on an Incucyte (Essen Biosciences). Migration was analyzed using the Incucyte software and migration was measured as relative wound density.

#### Transferrin uptake

50000 cells/well were seeded in at least two 24 well plates (1 plate to measure uptake and the other to measure surface receptor). On the next day, cells were incubated at 37°C for 8 min with pre-warmed serum-free media containing 10 μg/ml Alexa Fluor-488-labeled human transferrin. After incubation, cells were washed with PBS, detached with 0.25% trypsin at 37°C, received ice-cold serum containing media, spun, washed with ice-cold PBS, fixed with 4% PFA for 10 min at 4°C, spun and resuspended in PBS. To measure surface transferrin receptor, cells were detached using Accutase (Sigma, A6964), washed with ice-cold PBS, incubated on ice for 45 min with serum-free media containing 10 μg/ml Alexa Fluor-488-labeled human transferrin, spun, washed twice with ice-cold PBS, fixed with 4% PFA for 10 min at 4°C, spun and resuspended in PBS. Cells were analyzed using a Sony iCyt Eclipse flow cytometer (Sony Biotechnology Inc) or in a LSRFortessa (BD bioscencies). At least 5000 cells for each replicate were measured for each experiment. Normalized transferrin uptake was calculated by diving the median fluorescence signal of the uptake measurement by the median fluorescence signal of the surface receptor measurement. To combine experiments from different days, the results are displayed as relative to the average uptake value of the controls for each experiment. Transferrin uptake and surface transferrin receptor measurements were always performed and measured in parallel. For the dominant negative experiments using ITSN1-SH3d (Figure 3H), we measured the fluorescence values for transferrin only for cells with high expression levels of the RFP-tagged constructs (Cells with RFP signal at least 100 times above background). The values for the uptake and surface transferrin were then treated and displayed the same way as described above.

#### Antibody feeding assay

Cells were incubated at 4°C for 40 min with serum-free media with an anti-active integrinß1 antibody (12G10) in PBS (1:100 dilution). After incubation, unbound antibody was removed with two PBS washes. Cells were released from endocytic block by adding pre-warmed media and incubation at 37°C for the indicated periods of time. At the end of the incubation period, surface antibody was removed by two 2 min washes with stripping buffer (0.5% acetic acid, 0.5 M NaCl) followed by one PBS wash and fixation (4% PFA, 10 min at 4°C). Cells were then blocked and permeabilised (PBS, 5% goat serum, 0.5% Triton X-100) followed by incubation with a Alexa Fluor-488-labeled secondary antibody and imaged. Uptake index was calculated by dividing the background-subtracted signal by cell area.

#### Electron microscopy

Cells were grown on MatTek glass-bottomed Petri dishes and fixed in 2.5% glutaraldehyde 2% paraformaldehyde in 0.1 M cacodylate buffer pH7.4 O/N at 4°C. Washed in buffer and postfixed in 1% osmium tetroxide for 1 h at 4°C. Dehydrated in an ascending ethanol series and embedded in CY212 resin. Ultrathin sections were stained with saturated aqueous uranyl acetate and Reynolds lead citrate and examined using a Tecnai Spirit EM (FEI) operated at 80 KV.

#### GST pull downs

GST-tagged constructs were expressed in Rosetta (DE3) *Escherichia coli* overnight at 18°C. Cells were harvested, resuspended in lysis buffer (20 mM Tris pH 8.5, 500 mM NaCl, 2 mM EDTA), lysed using a Constant Cell Disruptor System (Constant Systems) and spun at 35000 rpm for 20 min at 4°C in a Beckman Ti45 rotor. The cleared supernatant was bound to glutathione beads for 1 h at 4°C. Beads were washed extensively with lysis buffer and resuspended in HEPES buffer (20 mM HEPES pH 7.5, 150 mM NaCl, 1 mM TCEP). Proteins bound to glutathione beads were used directly for pull downs. Rat brain lysates were incubated with protein-bound beads for 1h at 4°C followed by 6 washes with lysis buffer. Recombinant proteins were incubated with protein-bound beads for 10 min at room temperature and also washed 6 times with lysis buffer. Samples were run on SDS-PAGE gels and either immunoblotted or Coomassie stained. Rat brain lysates were prepared as follows: One brain was defrosted on ice and homogenized in a 15 mL Teflon-glass homogenizer with 4 mL of homogenization buffer (150 mM NaCl, 20 mM HEPES pH 7.5, 2 mM DTT, 1/1000 protease inhibitor cocktail and 0.1% Triton X-100). The lysate was cleared by centrifugation at 50000 rpm in a Beckman TLA 100.4 rotor.

#### Protein purification

FCHSD2 proteins used for crystallography, CryoEM, lipid binding and actin polymerization experiments were all expressed from constructs with an N-terminal His-SUMO tag. Proteins were expressed in Rosetta (DE3) *Escherichia coli* overnight at 18°C. Cells were harvested, resuspended in lysis buffer (20 mM Tris pH 8.5, 500 mM NaCl, 20 mM Imidazole), lysed using a probe sonicator and spun at 35000 rpm for 20 min at 4°C in a Beckman Ti45 rotor. The cleared supernatant was passed in a HisTrap FF column (GE healthcare) and extensively washed with lysis buffer. Bound protein was eluted with elution buffer (20 mM Tris pH8.5, 100 mM NaCl, 200 mM Imidazole) directly into an anion exchange column (HiTrap Q - GE healthcare). A Tris buffered salt gradient (100 mM to 500 mM NaCl) was used for elution and fractions containing the protein of interest were incubated overnight at 4°C with Sumo protease to cleave the His-SUMO tag. Proteins were further purified using a Superdex 200 (GE healthcare) size exclusion column using gel filtration buffer (150 mM NaCl, 20 mM HEPES pH 7.5, 500 μM TCEP). SNX9, CIP4 and cdc42 were purified using the same protocol, with the exception of the anion exchange step. FCHSD2 BAR-sfGFP used for NTA was purified using the protocol above with the exception of the anion exchange and the tag removal steps. N-WASP was purified as an N-terminal 6xHis tagged protein from insect cells (SF9) using the same protocol as described above for *E. coli* proteins with the exception of the step used to cleave the tag.

#### Liposome preparation

Liposomes were made by pore extrusion. Lipid mixtures dissolved in methanol were dried in glass tubes by Argon gas, rehydrated into buffer (20 mM HEPES pH 7.5, 150 mM NaCl) and filtered 20x through 0.8 μm diameter polycarbonate membranes (Nucleopore). Folch lipids used were a mixture of 1:1 between Sigma (B1502) and Avanti (131101P) brain extract lipids. Defined lipid mixtures were based on a mixture of 55:20:10, PC:PE:Cholesterol (molar ratio) supplemented with PS or Phosphatidylinositols at 10 or 5 parts, respectively.

#### Liposome binding assay

Purified protein (1 μM) was incubated with 125 μg/ml of liposomes for 15 min at room temperature and then spun down in a benchtop ultracentrifuge (Optima TL Ultracentrifuge) for 15 min at 80000rpm (TLA 100 rotor). Resuspended pellets and supernatants were analyzed by SDS-PAGE.

#### Actin polymerization assay

Actin was purified from muscle acetone powder as described previously (Doolittle et al., 2013). Pyrene-labeled actin (AP05) and ARP2/3 complex (RP01P) were purchased from Cytoskeleton Inc. Actin polymerization assays were performed in 40 μl reactions using 96 well half-area plates. Reactions were started by adding actin to a mix of all other components and actin polymerization buffer (10x buffer: 500 mM KCl, 20 mM MgCl_2_, 10 mM ATP). Fluorescence was measured in a Tecan Safire2 fluorescence plate reader using excitation/emission wavelength 365 nm (±20 nm) / 407 nm (±12 nm). All actin polymerization reactions were performed using 3 μM actin, 25 nM ARP2/3 and 50 nM N-WASP. Liposomes were used at 12.5 μM. To load cdc42 with GTPγS, purified cdc42 was incubated with 1.25 mM EDTA and 4 mM GTPγS for 45 min on ice. GTPγS was then locked in place by adding 4 mM extra MgCl_2_.

#### SEC- MALS

Size exclusion chromatography – multi angular light scattering (SEC-MALS) was performed using a Wyatt Heleos II 18 angle light scattering instrument coupled to a Wyatt Optilab rEX online refractive index detector. Detector 12 was replaced with Wyatt’s QELS detector. Samples for analysis were resolved on a Superdex S-200 10/300 analytical gel filtration column (GE Healthcare) running at 0.5 ml/min in gel filtration buffer (150 mM NaCl, 20 mM HEPES pH 7.5, 500 μM TCEP) before passing through the light scattering and refractive index detectors in a standard SEC-MALS format. Protein concentration was determined from the excess differential refractive index based on 0.186 ΔRI for 1 mg/ml. The concentration and the observed scattered intensity were used to calculate the absolute molecular mass from the intercept of the Debye plot using Zimm’s model as implemented in Wyatt’s ASTRA software.

#### Nanoparticle Tracking Analysis

Nanoparticle Tracking Analysis (NTA) was performed as described in (A.C., L.A.-S., and H.T.M., unpublished data). Measurements were taken on a Nanosight LM10 (Malvern) equipped with a 488nm laser, a 500nm long pass filter, a CMOS camera and a syringe pump. 800nm extruded liposomes (38:25:20:15:2, DOPC:PE:PS:Cholesterol:PI(3,4)P_2_ molar ratios) were diluted to a concentration of 2-8 x10^8^ particles/ml (∼1 μg/ml lipids) and FCHSD2-BAR-sfGFP was used at 1nM. 120 s movies at 25 frames per second were recorded under flow from the syringe pump (flow setting 50) to reduce bleaching and the tracking and analysis was performed using the Nanosight NTA software version 3.1 (Malvern). The size distribution of the total liposome population was obtained from movies measuring total diffracted light while the size distribution of FCHSD2-BAR-sfGFP was obtained from movies measuring only the emitted light from excited sfGFP detected with the 500 nm long pass filter.

#### CCV purification

Clathrin-coated vesicles (CCVs) were purified from HEK 293T cells following the protocol described in Girard et al. (2005). A simplified protocol flow chart is shown in Figure S6A. Briefly, cells were harvested from 5 confluent 15 cm dishes in buffer A (100 mM MES pH 6.5, 1 mM EGTA, 0.5 M MgCl_2_) and lysed by 20 strokes with a glass-teflon homogenizer. After centrifugation at 17000x g for 20 min, soluble proteins and vesicles (S1) were recovered and centrifuged at 56000xg for 1h to separate a crude microsomal fraction from soluble proteins (S2). The microsome pellet was resuspended in buffer A, layered in a Sucrose (8% w/v) Deuterium oxide cushion and centrifuged at 116000x g for 2 h to obtain a pellet of purified CCVs.

#### Crystallography

For crystallization, FCHSD2 SH3-2 and ITSN1 SH3d proteins were mixed in a 1:1 molar ratio at a final concentration of 38mg/ml. Crystallization trials were performed by sitting drop vapor diffusion in 200 nl drops (100 nl protein + 100 nl mother liquor) at 18°C. Crystals used for data collection grew in 0.5 M ammonium sulfate, 10% Glycerol, Tris pH 8.5. Crystals were cryoprotected in 25% glycerol. Data was collected at the Diamond Light Source (line i04-1) at a wavelength of 0.9282 Å. Datasets were integrated using MOSFLM (Battye et al., 2011) and scaled using SCALA (Evans, 2006). Structure was solved by molecular replacement with PHASER-MR (McCoy, 2007) using the NMR structures of FCHSD2 SH3-2 (PDB: 2DL7) and ITSN1-SH3d (PDB: 1UE9) as search models. The structure was solved in the space group I2_1_3 with 8 chains (4 dimers) in the asymmetric unit. The structure was refined by iterative cycles of manual model building in Coot (Emsley et al., 2010) and refinement with Refmac (Murshudov et al., 2011). The statistics for data processing and refinement are shown in Table S1. Molecular graphics were produced in CCP4MG (McNicholas et al., 2011).

#### Single particle CryoEM

For cryo-EM analysis, 3 μL of the sample (F2B1) at 0.15 mg/ml to 0.3 mg/ml were applied to Quantifoil R 1.2/1.3 Au 300 mesh grids, blotted for 3 – 3.5 s and vitrified in liquid ethane in an FEI Vitrobot MKIII, at 100% humidity at 4°C. Data were collected with a Titan Krios electron microscope (FEI) operated at 300 kV equipped with a K2 Summit direct electron detector (Gatan) mounted after a Gatan Imaging Filter (GIF) with a 20-eV slit. 20-frame image stacks were collected in electron-counting mode with a flux of 2 e^-^/Å^2^/s and a total dose of 40 e^-^/Å^2^ and a calibrated pixel size of 1.1 Å. Frames were aligned and averaged with MOTIONCORR (Li et al., 2013). Contrast-transfer-function parameters were calculated with Gctf (Zhang, 2016). All subsequent particle picking and data processing was done in a pre-release of Relion 2.0 (Scheres, 2012). From 766 micrographs, Relion autopick selected 230954 particles using as a reference 2D averages derived from a manually picked particle set. Autopicked particles were used for reference free 2D classification. The best 2D classes, containing 30270 particles, were selected for further processing. 3D classification generated classes that looked very similar to each other. Therefore, we used all particles from the best 2D classes for 3D refinement using a 40 Å low-pass filtered FES F-BAR structure (pdb 4DYL) as an initial model and applying C2 symmetry. Molecular graphics were produced in Chimera (Pettersen et al., 2004).

### Quantification and Statistical Analysis

Statistical analysis was performed with Prism 6.0. Student’s t test was used for pairwise comparisons. Tukey’s multiple comparisons test was used to determine significant differences between multiple samples. Data are presented as mean ± SD or SEM as indicated in the figure legends. Significance levels used were as follows: ^∗^p < 0.05, ^∗∗^p < 0.01, ^∗∗∗^p < 0.001.

### Data and Software Availability

The accession number for the FCHSD2 SH3-2/ITSN1 SH3d crystal structure reported in this paper is PDB: 6GBU. The accession number for the cryo-EM map for the FCHSD2 F-BAR reported in this paper is EMDB: EMD-4371.

## Supplemental Information

Table S1. Data Collection and Refinement Statistics, Related to Figure 3
