# Supplementary material for: A Flat BAR Protein Promotes Actin Polymerization at the Base of Clathrin-Coated Pits
Source: Cell. 2018 Jul 12;174(2):325–337.e14. doi: 10.1016/j.cell.2018.05.020 (PMC6057269; doi:10.1016/j.cell.2018.05.020)
Supplement: Table S1. Data Collection and Refinement Statistics, Related to Figure 3 [file mmc1.pdf]

Cell, Volume *174*

Supplemental Information

A Flat BAR Protein Promotes Actin Polymerization at the Base of Clathrin-Coated Pits

Leonardo Almeida-Souza, Rene A.W. Frank, Javier García-Nafría, Adeline Colussi, Nushan Gunawardana, Christopher M. Johnson, Minmin Yu, Gillian Howard, Byron Andrews, Yvonne Vallis, and Harvey T. McMahon

Table S1 - Data collection and refinement statistics.
Related to Figure 3

**Data collection**	
Space group	$I2_13$
Cell dimensions	
a, *b*, *c* (Å)	186.98
α, β, γ (°)	90
Resolution (Å)	132.2-3.44 (3.53-3.44)
R_{merge}	0.053 (0.936)
$I / \sigma I$	27.10 (2.00)
Completeness (%)	99.4 (94.1)
Redundancy	8.4 (7.9)
**Refinement**	
Resolution (Å)	132.22-3.44 (3.53-3.44)
No. reflections	13008
R_{work} / R_{free}	20.79 / 24.67
No. atoms	
Protein	3581
B-factors	
Protein	150
R.m.s.d.	
Bond lengths (Å)	0.011
Bond angles (°)	1.512
Ramachandran Plot Statistics (%)	
Favored	95.3
Allowed	4.7
Disallowed	0

[a]Values in parentheses are for highest-resolution shell.
